# Projected effectiveness of mandatory industrial fortification of wheat flour, milk, and edible oil with multiple micronutrients among Mongolian adults

**DOI:** 10.1371/journal.pone.0201230

**Published:** 2018-08-02

**Authors:** Sabri Bromage, Davaasambuu Ganmaa, Janet Wilson Rich-Edwards, Bernard Rosner, Jorick Bater, Wafaie Wahib Fawzi

**Affiliations:** 1 Department of Nutrition, Harvard University T.H. Chan School of Public Health, Boston, Massachusetts, United States of America; 2 Department of Medicine, Brigham and Women’s Hospital and Harvard Medical School, Boston, Massachusetts, United States of America; 3 Department of Epidemiology, Harvard University T.H. Chan School of Public Health, Boston, Massachusetts, United States of America; 4 Connors Center for Women’s Health and Gender Biology, Brigham and Women’s Hospital, Harvard Medical School, Boston, Massachusetts, United States of America; 5 Department of Biostatistics, Harvard University T.H. Chan School of Public Health, Boston, Massachusetts, United States of America; 6 Department of Global Health and Population, Harvard University T.H. Chan School of Public Health, Boston, Massachusetts, United States of America; Zhejiang University College of Biosystems Engineering and Food Science, CHINA

## Abstract

Industrial fortification of wheat flour is a potentially effective strategy for addressing micronutrient deficiencies in Mongolia, given its ubiquitous consumption and centralized production. However, Mongolia has not mandated fortification of any foods except for salt with iodine. This study modeled the effectiveness and safety of mandatory industrial fortification of wheat flour alone and in combination with edible oil and milk in reducing the prevalence of multiple micronutrient intake deficiencies among healthy non-pregnant adults in Mongolia. Six days of diet records (3 summer, 3 winter) were collected from 320 urban and rural adults across the country and analyzed for food and nutrient consumption using a purpose-built food composition table, and the Intake Monitoring and Planning Program (IMAPP) was used to project the effects of fortification on summer and winter bioavailable micronutrient intake and intake deficiency under different fortification guidelines within population subgroups defined by urban or rural locality and sex. Projections showed that flour fortification would be effective in reducing intake deficiencies of thiamin and folate, while marginal benefits of fortification with iron and riboflavin would be smaller given these nutrients’ higher baseline consumption, and fortification with zinc, niacin, and vitamin B12 may be unnecessary. Fortification of flour, oil, and milk with vitamins A, D, and E at levels suggested by international guidelines would substantially reduce vitamin A intake deficiency and would increase vitamin D intake considerably, with the greatest benefits elicited by flour fortification and smaller benefits by additionally fortifying oil and milk. These results support mandatory industrial fortification of wheat flour, edible oil, and milk with iron, thiamin, riboflavin, folate, and vitamins A, D, and E in Mongolia. Considerations will be necessary to ensure the fortification of these nutrients is also effective for children, for whom the potential benefit of zinc, niacin, and vitamin B12 fortification should be assessed.

## Introduction

Mongolians are traditionally a nomadic people who subsisted almost entirely on animal source foods throughout most of their history, owing to Mongolia’s poor soil quality and a cold, windy, and dry climate that makes it difficult to grow crops [1]. Although wheat flour has become much more important in recent centuries, the modern Mongolian diet still consists largely of dairy products and red meat, and little fruits, non-tuberous vegetables, legumes, nuts and seeds, and seafood [2,3]. Despite steady and impressive progress in curbing wasting, stunting, and low birth weight, a persisting lack of diversity in the Mongolian diet underlies a high prevalence of multiple biochemical micronutrient deficiencies among women and children, including those of iron, vitamin A, and vitamin D [4,5]. Micronutrient deficiencies in women of reproductive age pose a threat to their health, as well as the health of their offspring during pregnancy and nursing, and may lead to severe and permanent physical and cognitive deficits [6,7]. Maternal and child supplementation and home-fortification programs have been implemented in Mongolia but have faced challenges achieving desired distribution and compliance, particularly among the more dispersed nomadic herders who still account for approximately one-third of the country’s population [5,8], while efforts to develop and diversify Mongolian agricultural production, the food supply, and the diet have been varyingly hampered by the country’s climate, remoteness, and population’s historic adherence to a pastoralist food culture [9,10].

Mongolia is currently at a formative stage in developing its national food fortification policy, with the exception of universal salt iodization, which has been an effective measure in reducing the prevalence of biochemical iodine deficiency in the country since its implementation in 1995 [11,12]. A program for industrial wheat flour fortification was under development with assistance from the Asian Development Bank (ADB) between 2004 and 2007 but was aborted due to a range of financial, economic, political, procurement-related, and technical concerns among the various stakeholders involved [13]. Nonetheless, wheat flour remains an attractive vehicle for mandatory fortification in Mongolia given its high consumption across all population groups (with 341 grams of wheat and wheat products available per capita per day in the food supply as of 2013 [14]), its highly-centralized production system (10 mills process approximately 90% of the country’s domestically-produced flour), and the fact that the milling industry gained familiarity and technical experience with industrial fortification as part of the discontinued ADB project [13]. An intervention study by Tazhibayev and colleagues demonstrated that industrial wheat flour fortification caused statistically significant improvements in plasma folic acid concentrations among Mongolian women of reproductive age (reducing the prevalence of biochemical deficiency by 32%) and non-statistically significant reductions in serum ferritin [15], while studies by our research group have shown that fortified milk is highly effective in raising serum vitamin D concentrations in Mongolian schoolchildren (with locally-fortified milk raising the mean serum 25-hydroxyvitamin D concentration by 12 ng/mL more than unfortified milk) [16]. Moreover, a large body of international evidence has demonstrated industrial fortification of staple foods with micronutrients, particularly when mandated by law and appropriately monitored, to be an effective, cost-saving, and safe strategy for improving nutrition in populations [17].

In 2017, a bill proposing mandatory fortification of selected foods with multiple micronutrients was introduced to the Mongolian parliament and approved for legislative review in the same year. The purpose of this paper is to broadly inform the regulations falling under the upcoming fortification law with respect to suggested fortification levels for wheat flour and two other potential vehicles (edible oil and milk). We did this by projecting the effects that industrial fortification would have on summer and winter bioavailable nutrient intake and prevalence of intake deficiency and over-sufficiency among different Mongolian adult subgroups under multiple fortification guidelines. Projections are based upon baseline data on population food and nutrient consumption collected by our group from 2012–2016 as part of a nationwide nutrition survey.

## Methods

### Dietary assessment and nutritional analysis of diet

From each of 8 regions of Mongolia (7 national provinces and the municipality of Ulaanbaatar), 10 healthy men and 10 healthy non-pregnant women aged from 22–55 years and living in different households were randomly sampled from two geographically-circumscribed areas: the urban area lying within the city limits of a provincial capital and the rural area approximately 1 hour’s drive outside one of several smaller district capitals (with the exception of Ulaanbaatar, which was divided into an urban and a peri-urban zone, both of which were considered as urban for the purpose of this analysis), providing a sample of 20 urban and 20 rural or peri-urban adults per region (320 participants in total). Participants provided written informed consent prior to enrollment, and ethical approval for this study was obtained from the Harvard University T.H. Chan School of Public Health Institutional Review Board (Protocol #: 21002; Submission #: CR-21002-05).

From 2012 to 2016, local medical and public health university students were trained to prospectively and unobtrusively collect paired summer and winter 3-day weighed diet records (6 days in total) from each participant, including measured masses of consumed portions, raw ingredients, and cooked dishes. Demographic and lifestyle variables including age, self-reported height and weight, ethnicity, education, supplement use, housing type, and worksite were also assessed by questionnaire. Details of the training and dietary assessment are described elsewhere [18]. Collected diet records were translated and entered electronically in Excel by a team of translators, coded by a team of trained analysts according to a uniform protocol and food list, and tabulated to produce total daily consumption of 673 distinct food items on each record day. The content of fortification vehicles (wheat flour, edible oil, and milk) in ingredients, single-ingredient food items, and complex dishes was obtained either by averaging information across collected recipe data or from published equivalency factors [19,20]. Total consumption of each nutrient and vehicle by each participant on each day was calculated. See S1 File (Section A) for more details on nutritional analysis of diet and S2 File for the dataset analyzed in this study, including nutrient and food vehicle intake calculated for each person-day.

### Descriptive statistics

Characteristics of the study population were tabulated for each of four population subgroups (urban male, urban female, rural male, and rural female). To help contextualize the sub-national penetration of industrial fortification, the median and associated 1000-sample bootstrap confidence intervals of daily summer and winter consumption of wheat flour, edible oil, and milk in each subgroup were estimated using the R package SPADE (Statistical Program to Assess Dietary Exposure) ([21]; see S1 File (Section B) for more details on statistical programs), which statistically corrects intake distributions for observed within-person (day-to-day) variation in intake. Medians were estimated using SPADE’s 1-part model which assumes dietary components to be consumed in a habitual (daily) rather than an episodic fashion, as verified by inspection of dietary data. To produce more nationally-representative estimates, SPADE models were weighted according to survey weights generated using the 2010 Population and Housing Census of Mongolia as the reference population, incorporating information on age group, sex, urban or rural area, and day of the week [22]. Additionally, to describe the sources of wheat flour, edible oil, and milk in the diet, the mean daily fraction of each vehicle’s consumption contributed by each of different consumed food groups was calculated for each population subgroup and season, and weighted using the survey weights described above.

### Outline and assumptions of fortification models

Effects of mandatory industrial food fortification were modeled using the IMAPP software (Intake Monitoring and Planning Program) ([23]; see S1 File (Section B) for more details). For each of the 2 seasons (summer and winter) and 4 population subgroups (defined by urban or rural area and sex), models were run to estimate baseline distributions of nutrient intake corrected for within-person-variation, and project the effects of adding iron, zinc, thiamin, riboflavin, niacin, folate, and vitamin B12 to wheat flour, vitamin E to edible oil, and vitamins A and D to wheat flour, edible oil, and milk (in which all combinations of one, two, or three vehicles were individually or simultaneously fortified with vitamins A and D). Milk consumed in rural areas was assumed to be entirely produced at home rather than industrially-processed, as supported by prevailing food consumption patterns in Mongolia [24]. Effects of industrial milk fortification were therefore not modeled for rural areas. See S1 File (Section C) for additional assumptions of fortification models.

For each model, IMAPP outputted information on the estimated baseline and projected post-fortification median of daily intake in each subgroup, and the prevalence of nutrient intake deficiency (%<EAR) and over-sufficiency (>%UL) estimated using the EAR cut-point method [25]. In the case of iron, the cut-point method cannot be accurately applied given iron’s logarithmic requirement distribution among pre-menopausal women. IMAPP therefore estimated the post-fortification prevalence of iron intake deficiency using the full-probability approach [25]. EARs and ULs were drawn from the Harmonized Nutrient Reference Intakes [26] and assigned to each participant based on their sex and age in years. In the case of vitamin A, ULs are defined separately for preformed and total vitamin A, the former of which consists of dietary retinol and its ester retinol palmitate (the fortificant modeled in this study). Because our dietary data distinguished between intake of retinol and carotenoids, fortification models were able to estimate vitamin A over-sufficiency according to the sum of baseline retinol intake and added retinol palmitate using the retinol-specific UL of 3000 μg/day).

### Selection of modeled fortification levels

For each combination of nutrient and vehicle, different fortificant concentrations (informally referred to as “levels”) were modeled according to fractions and multiples of, or ranges otherwise suggested by levels found in six national (Mongolian), regional, and international guideline documents [27–32] (Table 1). In the case of iron, World Health Organization (WHO) international flour fortification guidelines suggest fortification levels for high or low flour extraction rates and 5 suitable chemical fortificants. A low extraction rate and iron in the form of ferrous fumarate were selected in accordance with Mongolian national wheat flour fortification guidelines [27].

**Table 1:** Fortification levels for wheat flour, edible oil, and milk.

			Fortification Levels Found in Published National, Regional, and International Reference Guidelines (per 100g of vehicle)		Fortification Levels Modeled in This Study (per 100g of vehicle)				
Nutrient	Fortificant	Vehicle	Published Guideline	Fortification Level	0	1	2	3	4
Thiamin	Thiamin mononitrate	Flour	Mongolian National Guideline [27]	0.4 mg	0.0 mg	0.2 mg	0.4 mg	0.6 mg	0.8 mg
Riboflavin	Riboflavin	Flour	Mongolian National Guideline [27]	0.4 mg	0.0 mg	0.2 mg	0.4 mg	0.6 mg	0.8 mg
Folate	Folic acid	Flour	Low WHO International Guideline [28]	100 μg	0 μg	100 μg	115 μg	130 μg	150 μg
			High WHO International Guideline [28]	130 μg					
			Mongolian National Guideline [27]	150 μg					
Vitamin B12	Cyanocobalamin	Flour	Low WHO International Guideline [28]	0.80 μg	0.00 μg	0.80 μg	0.87 μg	0.93 μg	1.00 μg
			High WHO International Guideline [28]	1.00 μg					
Iron	Ferrous fumarate	Flour	Low WHO International Guideline [28]	2.0 mg	0.0 mg	2.0 mg	2.3 mg	2.7 mg	3.0 mg
			High WHO International Guideline [28]	3.0 mg					
			Mongolian National Guideline [27]	3.0 mg					
Zinc	Zinc oxide	Flour	Low WHO International Guideline [28]	3.0 mg	0.0 mg	3.0 mg	3.3 mg	3.7 mg	4.0 mg
			High WHO International Guideline [28]	4.0 mg					
Vitamin E	Alpha tocopherol	Oil	Minimum DSM International Guideline [32]	6.5 mg	0.0 mg	6.5 mg	10.7 mg	14.8 mg	19.0 mg
			Maximum DSM International Guideline [32]	19.0 mg					
Niacin	Nicotinamide	Flour	Mongolian National Guideline [27]	3.0 mg	0.0 mg	1.5 mg	3.0 mg	4.5 mg	6.0 mg
Vitamin A	Retinol palmitate	Flour	Low WHO International Guideline [28]	100 μg	0 μg	100 μg	117 μg	133 μg	150 μg
			High WHO International Guideline [28]	150 μg					
		Oil	World Food Programme International Guideline [29]	900 μg	0 μg	450 μg	900 μg	1350 μg	1800 μg
		Milk	U.S. Food and Drug Administration National Guideline [31]	62 μg	0 μg	31 μg	62 μg	93 μg	124 μg
Vitamin D	Cholecalciferol	Flour	GCC Standardization Organization Regional Guideline [30]	55 IU	0 IU	28 IU	55 IU	83 IU	110 IU
		Oil	World Food Programme International Guideline [29]	300 IU	0 IU	150 IU	300 IU	450 IU	600 IU
		Milk	U.S. Food and Drug Administration National Guideline [31]	42 IU	0 IU	21 IU	42 IU	63 IU	84 IU

Low and High WHO International Guidelines are intended to be followed in countries consuming 300+ and 150–300 g/capita/day of wheat flour, respectively. Modeled fortification levels 0–4 are derived as fractions and multiples of, or ranges otherwise suggested by levels found in national, regional, and international reference guidelines. See Methods for modeled fortificant bioavailabilities. Abbreviations: IU (international unit; 40 IU = 1 μg), GCC (Gulf Cooperation Council), WHO (World Health Organization).

In the case of iron, zinc, vitamin A, folic acid, and vitamin B12, WHO guidelines suggest higher or lower fortification levels depending on whether national wheat flour availability is 150–300 or 300+ g/day, respectively. According to national food balance data, the Mongolian food supply contains 341 g/capita/day of wheat and wheat flour products [14]. In this study, weighted median summer and winter flour consumption across the four subgroups studied ranged from 189–336 g/day, and in prior analyses (not shown) we obtained nationally-weighted estimates of 207 and 310 g/day from a 2013 dietary assessment and household survey, respectively, collected by the Mongolian University of Science and Technology [33], and 277 g/day from analysis of pooled 2012 and 2014 waves of the Mongolian Socio-Economic Survey conducted by the National Statistical Office [34]. Given the different conclusions that may be drawn depending on the strictness with which one interprets “wheat flour availability”, the error associated with national food balance estimates, and the fact that true availability may in fact be close to around 300 g/capita/day, both high and low WHO estimates were considered in addition to Mongolian national guidelines in selecting the ranges of modeled fortification levels for iron, zinc, vitamin A, folic acid, and vitamin B12.

### Estimation of fortificant losses and overage factors

Projected effects of each fortification level were modeled three times, first allowing for predicted losses of fortificants during food processing, storage, and cooking, second incorporating overage factors to account for losses during processing and storage, and third (if theoretical cooking losses were non-negligible for the particular nutrient-vehicle combination) incorporating overage for processing, storage, and cooking losses (these three classes of models are indicated as “None” (no overage applied), “PS”, and “PSC”, respectively, in Supporting Tables). For readability, only projections which assume maximum (PSC) overage is included in the manuscript, while projections under all overage guidelines are given in Supporting Tables. To help inform a national industrial fortification program in Mongolia, nutrient-specific overage factors (factors by which fortification levels should be multiplied to compensate for food processing, storage, and cooking) were calculated for each food vehicle (S1 Table). See S1 File (Section D) for details on these calculations.

### Estimation of optimal fortification levels

In addition to models parameterized according to pre-specified fortification guidelines, an alternate set of models was run for each combination of subgroup, season, and nutrient to estimate subgroup- and season-specific optimal wheat flour fortification levels for adults, which, assuming overage for processing, storage, and cooking, would project a low target prevalence of bioavailable intake deficiency (this analysis excluded vitamin E, flour fortification of which was not considered in this study due to a lack of identified guidelines). The target prevalence was set at 5% for all micronutrients except vitamin D (while 2.5% is the target prevalence used to develop recommended dietary allowances and reference nutrient intakes, a doubly conservative value of 5% was selected under the reasoning that industrial fortification alone is not intended to entirely eliminate micronutrient deficiencies in a population). In the case of vitamin D, a more practical target of 50% was selected given the extremely high baseline prevalence of intake deficiency observed in the study population. For all nutrients except iron, the optimal levels were estimated automatically using an iterative process of incremental fortificant addition in IMAPP, termed “closing the gap”. Because this process is unable to accommodate iron’s lognormal requirement distribution, optimal levels for iron were instead estimated by manually modeling incremental additions until the target intake deficiency prevalence (5%) was reached based on the full-probability approach. If baseline intake deficiency prevalence of a particular nutrient in a particular subgroup and season was estimated at or below 5%, an optimal level was not estimated but instead simply taken to be 0. Optimal levels were also estimated for edible oil and milk, but were considered too monumental in comparison with published guidelines to be feasible for use in an actual fortification program, and are thus omitted from the results.

Estimated optimal levels were qualitatively compared to published reference guidelines to determine how appropriate the latter would be for application in Mongolia, and the effects of subgroup- and season-specific optimal levels on the prevalence of intake deficiency and over-sufficiency were modeled for both sexes in the same urban or rural area and season. Although optimal levels were derived based on assumptions of maximal overage (for processing, storage, and cooking losses), the effects of these levels were nonetheless modeled under all three overage scenarios considered in analysis of pre-specified fortification guidelines (i.e. no overage, overage for processing and storage losses, and overage for processing, storage, and cooking losses), in order to simulate the effect of considering or neglecting to consider overage in estimating optimal fortification levels (as with projections based on pre-specified fortification levels, only projected effects under maximum overage guidelines are presented in the manuscript, while effects projected under all overage guidelines are given in Supporting Tables).

### Additional modeled parameters

In addition to the parameters described previously, fortification models also incorporated data on the following:

- Bioavailability of each chemical fortificant stipulated by fortification guideline documents, relative to either the form of the nutrient found in food or to the most bioavailable form, as specified in intake guidelines: ferrous fumarate-18%, zinc oxide-50%, retinol palmitate-90%, folic acid-166.7%, cyanocobalamin-200%, cholecalciferol, alpha-tocopherol, thiamin mononitrate, riboflavin, and nicotinamide-100%. Bioavailabilities were informed by the literature as well as the average meat consumption and phytate:zinc molar ratio observed in the study population [17].
- The fraction of milk consumed in urban areas which is industrially produced and thus able to be industrially fortified (20.8%), estimated by dividing the estimated fraction of national milk production currently subject to industrial processing (10.3%) by the fraction of national milk consumption accounted for by urban households (49.3%), assuming no industrially-processed milk is consumed in rural areas [24,34,35].
- A moderate prevalence of modern contraceptive use (including oral contraceptives) among women of reproductive age (48.2%) [36], incorporated in modeling the effects of iron fortification.

## Results

### Population characteristics

Urban participants in this study population were, on average, younger (mean age in urban vs. rural areas: 37.7 ± 9.7 vs. 40.6 ± 9.4 years, respectively), more ethnically homogenous (88% vs. 78% Khalkh Mongolian), more formally educated (14.2 ± 2.8 vs. 10.1 ± 3.7 years), and more likely to consume multivitamins than their rural counterparts (11% vs. 5%), although multivitamin use (expressed as “ever in past 12 months”) was assumed to be uncommon enough in both groups so as not to substantially affect the distribution of baseline nutrient intake (Table 2). Mean body mass index of urban and rural participants was 25.9 ± 4.1 and 25.1 ± 3.7 kg/m^3^, respectively. Urban participants consisted mostly of office workers (80%) dwelling in a variety of housing types, while rural participants were almost exclusively nomadic herders (99%) living primarily in traditional yurts (94%). The availability of diet records data was 100% in summer and 90 to 94% for rural and urban participants, respectively, in winter. In total, 1,839 person-days of intake data were available for analysis from 320 participants (5.75 days/person).

**Table 2:** Characteristics of study population.

Characteristic	Rural (n = 140)	Urban (n = 180)
Female sex, n (%)	70 (50)	90 (50)
Age (years), mean (SD)	40.6 (9.4)	37.7 (9.7)
BMI (kg/m^2^), mean (SD)	25.1 (3.7)	25.9 (4.1)
Ethnicity, n (%)		
Khalkh	78 (72)	143 (88)
Zakhchin	24 (22)	13 (8)
Other	6 (6)	6 (4)
Education (years), mean (SD)	10.1 (3.7)	14.2 (2.8)
Multivitamin use, n (%) *	7 (5)	19 (11)
Housing, n (%) **		
Yurt	102 (94)	28 (18)
Apartment	1 (1)	42 (26)
House (centrally-heated)	2 (2)	49 (31)
House (no central heating)	3 (3)	41 (26)
Worksite, n (%)		
Outdoor labor	0 (0)	21 (12)
Office	2 (1)	143 (80)
Herder	138 (99)	0 (0)
Factory	0 (0)	6 (3)
Other	0 (0)	9 (5)
Data available for analysis, n (%)		
Summer diet records	140 (100)	180 (100)
Winter diet records	132 (94)	162 (90)

Values represent n (%) or mean (SD). Percentages are calculated after excluding missing values. BMI: body mass index.

* Multivitamin use expressed as “Ever during the past twelve months”.

** Housing heating type assessed as an indicator of socioeconomic status.

### Consumption and dietary sources of fortification vehicles

Median consumption of wheat flour was highest among rural males in winter (325 g/day), lowest among urban and rural females (range: 183–231), and displayed a seasonal pattern in rural areas such that median winter consumption exceeded summer’s by 69 and 46 g/day in males and females, respectively (S1 Fig). Median consumption of edible oil was lowest among rural females in summer (10.3 g/day), highest among rural males in winter (17.6 g/day), and in urban areas, summer consumption exceeded winter's by 3.4 and 3.8 g/day in males and females, respectively. Median milk consumption was higher in rural areas (range across sexes and seasons: 122–210 g/day) than urban areas (57–69 g/day), and in rural areas displayed a seasonal pattern opposite to that of wheat flour such that median summer consumption exceeded winter’s by 42 and 58 g/day in men and women, respectively. Major contributors of dietary wheat flour included (1) steamed, fried, or boiled dumplings (mean percentage of dietary wheat flour across seasons and subgroups: 22.3%), (2) tsuivan, a dish of steamed wheat-flour noodles and stir-fried meat (20.9%), (3) bread and bread with toppings or condiments (20.1%), (4) soups (17.9%), and (5) baked or fried flour products, excluding bread (12.9%). Edible oil was consumed primarily from (1) tsuivan (33.4%), (2) boortsog, a deep-fried wheat flour snack similar to a donut (24.6%), (3) huurga, a broad category of meat-based dishes made with various stir-fried and steamed ingredients (20.8%), and (4) dumplings (11.5%) (S2 Fig). The vast majority of milk was consumed in the form of boiled milk, milk tea, and milk-based soups.

### Fortification of wheat flour with thiamin, riboflavin, folic acid, and vitamin B12

A summary of baseline and projected (post-fortification) prevalence of intake deficiency for all 10 nutrients analyzed is given in S2 Table and is accompanied by a Policy Brief in S3 File (Mongolian versions of the results summary and policy brief are given in S4 File).

Projected results of wheat flour fortification with four vitamins for which upper limits are not established (thiamin, riboflavin, folate, and vitamin B12) are given in Table 3 (under maximum overage guidelines) and S3 Table (under all overage guidelines). At baseline, riboflavin intake deficiency is uncommon (range of deficiency prevalence among 8 season-subgroups: 0.6–11.4%) and deficiency of vitamin B12 is almost non-existent. Implementing Level 1 riboflavin fortification, equal to half the level suggested in national wheat flour fortification guidelines published by the Mongolian University of Science and Technology (MUST), would, with maximum overage for processing, storage, and cooking, reduce this range to 0.0–4.6%. By contrast, thiamin and folate intake deficiencies are highly prevalent at baseline (range: 36.2–81.9% and 94.4–100.0%, respectively). Level 2 fortification (equal to the local MUST guideline) with maximum overage would reduce the range of thiamin and folate deficiency prevalence to 0.1–14.7% and 0.1–22.1%, respectively, leaving urban females with the only projected deficiency prevalence of thiamin above 4.4% and folate above 7.4% in either season.

**Table 3:** Prevalence of thiamin, riboflavin, folate, and vitamin B12 intake deficiency.

		Rural Females		Rural Males		Urban Females		Urban Males	
Nutrient	Fortification Level	Summer	Winter	Summer	Winter	Summer	Winter	Summer	Winter
Thiamin	0	65.0	81.9	36.2	53.4	58.4	73.5	53.3	62.5
	1	14.3	23.2	9.2	2.4	19.0	34.1	9.5	15.0
	2	1.2	4.4	2.6	0.1	5.8	14.7	1.0	2.1
	3	0.0	1.0	1.0	0.0	2.0	6.9	0.1	0.3
	4	0.0	0.3	0.4	0.0	0.8	3.5	0.0	0.0
Riboflavin	0	3.4	10.9	5.3	0.6	8.0	11.4	11.2	4.8
	1	0.3	1.3	1.2	0.0	2.3	4.6	2.1	0.6
	2	0.0	0.2	0.5	0.0	0.8	1.8	0.5	0.0
	3	0.0	0.0	0.2	0.0	0.3	0.9	0.1	0.0
	4	0.0	0.0	0.2	0.0	0.1	0.5	0.0	0.0
Folate	0	100.0	99.3	97.1	97.1	99.7	99.0	98.0	94.4
	1	12.3	12.4	6.3	0.3	18.7	29.4	1.0	1.4
	2	3.9	7.4	4.3	0.1	13.1	22.1	0.3	0.6
	3	1.2	4.4	2.8	0.0	9.3	16.8	0.1	0.3
	4	0.2	2.4	1.5	0.0	5.8	11.6	0.0	0.1
Vitamin B12	0	0.0	0.0	0.0	0.0	0.5	0.0	0.0	0.0
	1	0.0	0.0	0.0	0.0	0.0	0.0	0.0	0.0
	2	0.0	0.0	0.0	0.0	0.0	0.0	0.0	0.0
	3	0.0	0.0	0.0	0.0	0.0	0.0	0.0	0.0
	4	0.0	0.0	0.0	0.0	0.0	0.0	0.0	0.0

Values represent the percentage of each subgroup’s nutrient intake lying below the subgroup-specific estimated average requirement (EAR) at baseline (Level 0) and projected under different fortification guidelines and maximum overage guidelines for processing, storage, and cooking. Shading indicates the extent of projected intake deficiency (0%: green; 50%: yellow; 100%: red). See Methods and Table 1 for description of levels and references.

### Fortification of wheat flour and edible oil with iron, zinc, vitamin E, and niacin

Projected results of wheat flour and oil fortification with four micronutrients for which upper limits are established (iron, zinc, vitamin E, and niacin) are given in Table 4 (under maximum overage guidelines) and S4 Table (under all overage guidelines). The baseline prevalence of iron intake deficiency is moderate among urban and rural females (range of deficiency prevalence among 4 season-subgroups: 10.6–22.2%; over-sufficiency prevalence: 0.0%) but not males (range of deficiency: 0.0–0.5%; over-sufficiency: 0.0–0.3%). Iron fortification of flour at Level 1 (the lower level suggested by the WHO guideline for Mongolia), ensuring maximum overage, would eliminate deficiency and project a modest increase in the prevalence of over-sufficiency among males (range: 0.0–1.3%), and would project a significant reduction in deficiency among females (range: 3.5–11.1%; over-sufficiency: 0.0–0.1%). The baseline prevalence of zinc intake deficiency and over-sufficiency are low (range of deficiency across 8 season-subgroups: 0.3–2.1%; over-sufficiency: 0.0–3.7%). Flour fortification at Level 1 (the lower WHO guideline for Mongolia), with maximum overage, would all but eliminate deficiency (range: 0.0–0.2%) while projecting a considerable increase in the prevalence of over-sufficiency among urban and rural males (range across 4 season-subgroups: 9.0–25.4%) and less so among females (range: 0.0–3.8%).

**Table 4:** Prevalence of iron, zinc, vitamin E, and niacin intake deficiency and over-sufficiency.

		Rural Females				Rural Males				Urban Females				Urban Males			
		Summer		Winter		Summer		Winter		Summer		Winter		Summer		Winter	
Nutrient (Vehicle)	Fortification Level	%< EAR	%> UL	%< EAR	%> UL	%< EAR	%> UL	%< EAR	%> UL	%< EAR	%> UL	%< EAR	%> UL	%< EAR	%> UL	%< EAR	%> UL
Iron (Flour)	0	18.7	0.0	10.6	0.0	0.5	0.0	0.0	0.0	16.8	0.0	22.2	0.0	0.2	0.0	0.0	0.3
	1	7.3	0.0	3.5	0.0	0.0	1.3	0.0	0.3	7.5	0.1	11.1	0.0	0.0	0.0	0.0	1.2
	2	5.7	0.0	2.9	0.0	0.0	1.8	0.0	0.5	6.8	0.2	9.8	0.1	0.0	0.1	0.0	1.5
	3	4.7	0.0	2.1	0.0	0.0	2.5	0.0	0.8	6.0	0.2	8.5	0.2	0.0	0.2	0.0	1.9
	4	3.9	0.0	1.9	0.0	0.0	3.2	0.0	1.2	5.1	0.3	8.0	0.2	0.0	0.3	0.0	2.7
Zinc (Flour)	0	1.4	0.0	1.9	0.0	0.8	3.7	0.5	1.6	2.1	0.4	0.3	0.3	0.5	0.4	0.8	2.9
	1	0.0	0.0	0.1	1.3	0.0	15.6	0.0	25.4	0.2	3.8	0.0	0.5	0.0	9.0	0.0	15.5
	2	0.0	0.0	0.0	2.0	0.0	18.8	0.0	30.6	0.1	4.9	0.0	0.8	0.0	11.9	0.0	18.4
	3	0.0	0.0	0.0	2.9	0.0	22.4	0.0	36.2	0.1	6.3	0.0	1.3	0.0	15.3	0.0	21.9
	4	0.0	0.0	0.0	3.9	0.0	26.4	0.0	42.0	0.1	7.8	0.0	1.8	0.0	19.1	0.0	25.4
Vitamin E (Oil)	0	100.0	0.0	99.7	0.0	98.8	0.0	99.8	0.0	98.8	0.0	99.0	0.0	100.0	0.0	99.5	0.0
	1	100.0	0.0	99.0	0.0	95.2	0.0	97.8	0.0	97.2	0.0	97.6	0.0	98.0	0.0	97.4	0.0
	2	100.0	0.0	98.1	0.0	91.8	0.0	93.6	0.0	95.0	0.0	96.1	0.0	93.8	0.0	94.7	0.0
	3	99.8	0.0	96.9	0.0	87.1	0.0	86.8	0.0	91.5	0.0	94.2	0.0	85.4	0.0	90.0	0.0
	4	99.4	0.0	95.1	0.0	81.4	0.0	77.9	0.0	86.7	0.0	92.0	0.0	72.5	0.0	84.8	0.0
Niacin (Flour)	0	11.6	0.0	13.0	0.1	3.4	6.1	0.5	15.4	10.9	1.2	13.4	0.2	2.2	6.8	2.0	12.7
	1	2.8	0.0	3.5	0.6	1.3	18.4	0.0	33.2	4.5	4.0	6.4	0.9	0.5	16.3	0.5	22.8
	2	0.5	0.2	0.8	2.8	0.6	34.4	0.0	57.2	2.0	9.9	3.2	3.8	0.1	31.9	0.1	36.2
	3	0.0	0.9	0.5	10.7	0.3	49.7	0.0	78.7	1.0	19.1	1.8	9.6	0.0	50.2	0.0	51.2
	4	0.0	3.8	0.2	22.9	0.2	63.7	0.0	90.9	0.5	30.1	1.1	18.2	0.0	67.0	0.0	65.4

Values represent the percentage of each subgroup’s nutrient intake lying below the subgroup-specific estimated average requirement (EAR) or above its upper limit (UL), respectively, at baseline (Level 0) and projected under different fortification levels and maximum overage guidelines for processing, storage, and cooking. Shading indicates the extent of projected intake deficiency or over-sufficiency (0%: green; 50%: yellow; 100%: red). See Methods and Table 1 for description of levels and references.

By contrast, baseline vitamin E intake deficiency prevalence is extremely high (range across 8 season-subgroups: 98.8–100%) with no prevalence of over-sufficiency. Oil fortification at Level 2 (the DSM international guideline), with overage for processing, cooking, and storage, would have a relatively small effect on deficiency prevalence (range across 8 subgroups and seasons: 91.8–100%) without affecting that of over-sufficiency. In the case of niacin, the baseline prevalence of intake deficiency and over-sufficiency are qualitatively opposite between males (range of deficiency prevalence across 4 seasons-subgroups: 0.5–3.4%; over-sufficiency: 6.1–15.4%) and females (deficiency: 10.9–13.4%; over-sufficiency: 0.0–1.2%). Flour fortification at Level 1 (half the local guideline), with maximum overage, would reduce the range of intake deficiency prevalence among males (0.0–1.3%) and females (2.8–6.4%) while considerably increasing that of over-sufficiency in males (16.3–33.2%) but not females (0.0–4.0%).

### Fortification of wheat flour, edible oil, and milk with vitamin A

Results of vitamin A and D fortification in rural areas are provided in the manuscript and Supporting Tables, while results for urban areas are given in a set of four interactive graphs hosted externally, links to which are referenced below. Captions to interactive graphs are given in S5 File and at http://rpubs.com/sbromage.

Flour and oil fortification, simultaneously or on their own, would substantially reduce the prevalence of vitamin A intake deficiency. Baseline vitamin A deficiency is common in urban areas (range across 4 season-subgroups: 46.4–73.4%; http://rpubs.com/sbromage/Vitamin_A_intake_deficiency) and rural areas (40.0–71.3%; Tables 5 and S5), while over-sufficiency is less common (urban: 0.0–14.3%; rural: 0.4–5.2%; http://rpubs.com/sbromage/Vitamin_A_over-sufficiency, Tables 5 and S5) (see note in next paragraph regarding over-sufficiency estimates for vitamin A). Flour fortification at Level 2 (116.7 μg retinol palmitate/100g flour), with maximum overage, would reduce these deficiency ranges to 14.3–41.8% and 5.2–40.8% in urban and rural areas, respectively (over-sufficiency: 0.2–10.0% and 0.6–4.6%, respectively), while Level 2 oil fortification (the level suggested in World Food Programme (WFP) international guidelines) would reduce deficiency to 26.9–56.4% and 26.9–61.2% (over-sufficiency: 0.1–8.3% and 0.0–5.1%). Together, flour and oil fortification at these guidelines would reduce the ranges of urban and rural deficiency prevalence to 8.3–30.0% and 0.6–24.9%, respectively (over-sufficiency: 1.2–9.7% and 0.1–5.3%). Fortification of milk alone would have a small effect on deficiency prevalence in urban areas: 45.1–71.8% (over-sufficiency: 0.0–13.8%); with oil: 26.5–55.2% (over-sufficiency: 0.1–7.3%); with flour: 13.1–40.6% (over-sufficiency: 0.2–9.7%); with flour and oil: 7.6–29.1% (over-sufficiency: 0.2–9.7%).

**Table 5:** Prevalence of vitamins A and D intake deficiency and over-sufficiency (rural areas).

		Vitamin A								Vitamin D							
		Females				Males				Females				Males			
		Summer		Winter		Summer		Winter		Summer		Winter		Summer		Winter	
Flour Fortification Level	Oil Fortification Level	%< EAR	%> UL	%< EAR	%> UL	%< EAR	%> UL	%< EAR	%> UL	%< EAR	%> UL	%< EAR	%> UL	%< EAR	%> UL	%< EAR	%> UL
0	0	40.0	0.6	71.3	0.4	49.1	2.8	41.5	5.2	100.0	0.0	100.0	0.0	100.0	0.0	100.0	0.0
	1	35.8	2.1	67.3	0.0	43.5	5.3	32.7	6.3	100.0	0.0	100.0	0.0	100.0	0.0	100.0	0.0
	2	30.5	1.3	61.2	0.0	35.6	5.1	26.9	2.2	100.0	0.0	100.0	0.0	100.0	0.0	100.0	0.0
	3	23.9	0.8	54.5	0.0	27.9	5.0	20.5	1.2	100.0	0.0	100.0	0.0	100.0	0.0	100.0	0.0
	4	18.2	0.6	44.7	0.0	21.1	5.1	15.0	0.7	100.0	0.0	100.0	0.0	100.0	0.0	100.0	0.0
1	0	17.0	1.6	45.5	1.4	26.4	4.5	9.1	0.9	100.0	0.0	100.0	0.0	99.8	0.0	100.0	0.0
	1	13.1	1.1	37.4	1.0	19.6	5.0	3.9	0.5	100.0	0.0	100.0	0.0	99.5	0.0	100.0	0.0
	2	9.5	0.8	29.9	0.7	14.0	5.2	1.5	0.2	100.0	0.0	100.0	0.0	98.9	0.0	100.0	0.0
	3	6.5	0.7	22.8	0.6	9.5	5.3	0.4	0.2	100.0	0.0	100.0	0.0	97.5	0.0	100.0	0.0
	4	4.3	0.4	15.8	0.5	6.5	5.5	0.1	0.0	100.0	0.0	99.9	0.0	95.1	0.0	99.5	0.0
2	0	13.3	1.5	40.8	1.4	22.5	4.6	5.2	0.6	100.0	0.0	100.0	0.0	96.5	0.0	100.0	0.0
	1	10.1	0.9	32.6	1.2	16.4	5.2	1.9	0.3	100.0	0.0	100.0	0.0	93.8	0.0	100.0	0.0
	2	7.0	0.8	24.9	0.8	11.2	5.3	0.6	0.1	100.0	0.0	99.9	0.0	89.7	0.0	99.1	0.0
	3	4.9	0.6	19.1	0.7	7.6	5.6	0.1	0.0	100.0	0.0	99.6	0.0	84.6	0.0	95.6	0.0
	4	3.2	0.3	13.6	0.6	5.1	5.8	0.0	0.0	100.0	0.0	98.5	0.0	78.5	0.0	88.8	0.0
3	0	10.2	1.3	35.6	1.5	19.0	4.8	2.5	0.3	100.0	0.0	99.6	0.0	83.1	0.0	96.5	0.0
	1	7.4	0.9	27.9	1.2	13.7	5.4	0.8	0.2	100.0	0.0	98.7	0.0	77.1	0.0	89.8	0.0
	2	4.7	0.7	21.1	0.9	9.2	5.5	0.2	0.0	100.0	0.0	97.0	0.0	70.3	0.0	79.1	0.0
	3	3.3	0.6	15.6	0.8	6.2	6.0	0.0	0.0	100.0	0.0	94.5	0.0	63.1	0.0	67.5	0.0
	4	2.0	0.3	10.7	0.6	4.3	6.2	0.0	0.0	99.8	0.0	90.8	0.0	55.2	0.0	55.9	0.0
4	0	7.6	0.3	31.1	1.5	15.9	5.0	1.0	0.2	100.0	0.0	93.3	0.0	61.0	0.0	63.2	0.0
	1	5.1	0.7	23.3	1.2	11.3	5.5	0.2	0.0	100.0	0.0	89.2	0.0	54.6	0.0	51.0	0.0
	2	3.4	0.6	17.8	1.0	7.5	5.9	0.0	0.0	99.8	0.0	84.8	0.0	47.9	0.0	40.3	0.0
	3	2.2	0.5	12.4	0.9	5.0	6.2	0.0	0.0	99.1	0.0	79.0	0.0	41.3	0.0	31.1	0.0
	4	1.3	0.3	8.4	0.8	3.4	6.6	0.0	0.0	97.7	0.0	73.6	0.0	35.1	0.0	24.0	0.0

Values represent the percentage of each rural subgroup’s nutrient intake lying below the subgroup-specific estimated average requirement (EAR) or above its upper limit (UL), respectively, at baseline (Level 0) and projected under different fortification levels and maximum overage guidelines for processing, storage, and cooking. Shading indicates the extent of projected intake deficiency or over-sufficiency (0%: green; 50%: yellow; 100%: red). See Methods and Table 1 for description of levels and references.

The results of most vitamin A fortification models project a paradoxical decrease in the prevalence of over-sufficiency as fortification levels increase. The summer and winter distributions of vitamin A intake within each of the 4 subgroups, particularly vitamin A from retinol, appeared significantly more right-skewed than those of any of the other 9 nutrients analyzed in this study. Extreme right-skew in vitamin A measurements is usually related to vitamin A’s high intra-person variation, which generally requires many more days of dietary data to be collected than other vitamins or minerals to allow a precise estimate of long-term intake [37]. In this population, in light of its relatively monotonous diet, skew is more attributable to high (but not highly variable within persons) consumption of organ meats and dairy products among certain consumers (particularly in rural areas). It is therefore likely that while IMAPP was capable of accurately drawing the left tail of baseline and projected vitamin A intake distributions in this study, it usually lacked sufficient statistical information to accurately draw the right tail. As a result, while estimates of vitamin A intake deficiency prevalence and median intake are estimated accurately, estimates of over-sufficiency prevalence are estimated poorly and should not serve as a basis for inference about the larger Mongolian adult population.

### Fortification of wheat flour, edible oil, and milk with vitamin D

At baseline, the population is wholly (100%) intake deficient in vitamin D (http://rpubs.com/sbromage/Vitamin_D_intake_deficiency, Tables 5 and S5). Simultaneous Level 2 fortification of wheat flour, edible oil, and (in urban areas) milk (the level suggested by Gulf Cooperation Council Standardization Organization (GSO) regional, WFP international, and FDA (Food and Drug Administration) national guidelines, respectively), would have a fairly modest effect on the range of deficiency prevalence in urban areas (range across 4 season-subgroups: 97.4–99.0%) and rural areas (89.7–100%). However, these measures would increase median intake dramatically. At baseline, median intake across 4 season-subgroups ranges from 32–52 IU/day in urban areas and 18–41 IU/day in rural areas (http://rpubs.com/sbromage/Vitamin_D_median_intake, Tables 6 and S6). Upon fortification of flour at Level 2 (the GSO regional guideline), these urban and rural ranges increase to 147–201 and 127–204 IU/day, respectively. Oil fortification at Level 2 (the DSM international guideline) would also be effective, increasing the ranges of urban and rural median intake to 79–116 and 52–97 IU/day, respectively, while the combination of Level 2 flour and oil fortification would result in urban and rural ranges of 154–263 and 149–253 IU/day, respectively. Level 2 milk fortification (the FDA national guideline) would be relatively ineffective in increasing median intake in urban areas: 31–58 IU/day; with flour: 133–208 IU/day; with oil: 62–122 IU/day; with both flour and oil: 161–269 IU/day. Neither at baseline nor after modeling Level 4 fortification of all three vehicles simultaneously (twice the levels suggested in the GSO, WFP, and FDA guidelines) is the prevalence of vitamin D over-sufficiency projected to exceed 0% in any of the 8 season-subgroups considered.

**Table 6:** Median intake of D (IU/day) (rural areas).

		Females		Males	
		Summer	Winter	Summer	Winter
Flour Fortification Level	Oil Fortification Level	IU/day	IU/day	IU/day	IU/day
0	0	29.1	18.2	41.1	31.3
	1	40.8	36.8	70.0	58.1
	2	52.1	53.2	97.2	82.8
	3	63.0	69.5	122.9	107.1
	4	73.8	85.3	148.7	131.6
1	0	78.5	82.0	123.4	119.5
	1	89.8	98.2	148.7	143.9
	2	100.8	114.4	174.7	167.7
	3	111.8	130.3	200.1	191.9
	4	122.6	146.3	225.7	215.8
2	0	127.3	144.0	202.2	204.6
	1	138.2	160.3	228.0	228.8
	2	149.3	176.2	253.4	252.7
	3	160.0	192.0	278.2	276.5
	4	171.2	207.8	303.8	300.3
3	0	175.9	206.1	281.8	289.5
	1	186.8	222.1	305.8	313.5
	2	197.8	238.1	330.3	337.2
	3	208.7	253.8	355.1	362.9
	4	219.7	269.0	381.7	386.7
4	0	224.3	268.0	360.2	374.2
	1	235.4	284.0	383.1	398.0
	2	246.3	300.3	407.7	421.9
	3	257.3	300.3	433.4	447.7
	4	268.2	300.3	459.9	472.1
Female Optimum	0	400.0	400.0	635.0	557.9
Male Optimum	0	252.1	285.5	400.0	400.0

Values represent the median intake of vitamin D (IU/day) in each rural subgroup at baseline (Level 0) and projected under different fortification levels and maximum overage guidelines for processing, storage, and cooking. Shading indicates the magnitude of projected median intake (minimum (18.2 IU/day): red; median (208.3 IU/day): yellow; estimated average requirement (400.0 IU/day): green). See Methods and Table 1 for description of levels and references, and Methods and Table 8 for description and specifications of male and female optimal levels. IU: international unit (40 IU = 1 μg).

### Optimal fortification levels for wheat flour

Tables 7 and 8 compare the range of published national, regional, and international wheat flour fortification levels (reproduced from Table 1) to the optimal fortification levels estimated for Mongolian adults in this study, for each nutrient, population subgroup, and season, and show how implementing these optimal levels would affect season-specific prevalence of intake deficiency and over-sufficiency among males and females in the same urban or rural areas under maximum overage guidelines (see S7 and S8 Tables for projected effects under all overage guidelines). Results of this analysis suggest that of all the nutrients considered in this study, the published national guideline for thiamin alone may be close to optimal for adults in Mongolia (assuming appropriate overage is applied). National guidelines for riboflavin and niacin, international guidelines for zinc and vitamin B12, and both national and international guidelines for iron may be too high (suggesting that fractions of published guidelines may be optimal for adults), while national and international guidelines for folate and vitamin A may be too low (suggesting that multiples may be optimal). Tables 8 and S8 also indicate that, although not intended for use in Mongolia, the fortification level suggested by the regional GSO guideline for vitamin D is also below the optimum for Mongolian adults. For certain nutrients there is a wide range in estimated optimal fortification levels across seasons and subgroups, including folate (range: 104–337 μg/100 g), vitamin A (106–267 μg/100 g), and vitamin D (118–209 IU/100 g).

**Table 7:** Optimal flour fortification levels for thiamin, riboflavin, and folate.

			**Area and Season: Rural Summer**			**Area and Season: Rural Winter**		
**Nutrient**	**Published Levels** **(per 100g of flour)**	**Modeled Optimum**	**Optimal Level (per 100g of flour)**	**%<EAR, Females**	**%<EAR, Males**	**Optimal Level (per 100g of flour)**	**%<EAR, Females**	**%<EAR, Males**
Thiamin	0.4 mg	Female Optimum	0.3 mg	4.8	5.2	0.4 mg	4.8	0.1
		Male Optimum	0.3 mg	4.3	4.9	0.2 mg	31.8	4.4
Riboflavin	0.4 mg	Female Optimum	0.0 mg	3.4	5.3	0.1 mg	4.7	0.0
		Male Optimum	0.0 mg	3.1	4.9	0.0 mg	10.9	0.6
Folate	100 μg, 130 μg, 150 μg	Female Optimum	187.0 μg	4.9	4.7	210.7 μg	5.0	0.0
		Male Optimum	183.2 μg	6.1	5.0	104.1 μg	42.1	5.0
Vitamin B12	0.80 μg, 1.00 μg	Female Optimum	0.0 μg	0.0	0.0	0.0 μg	0.0	0.0
		Male Optimum	0.0 μg	0.0	0.0	0.0 μg	0.0	0.0
			**Area and Season: Urban Summer**			**Area and Season: Urban Winter**		
**Nutrient**	**Published Levels (per 100g of flour)**	**Modeled Optimum**	**Optimal Level (per 100g of flour)**	**%<EAR, Females**	**%<EAR, Males**	**Optimal Level (per 100g of flour)**	**%<EAR, Females**	**%<EAR, Males**
Thiamin	0.4 mg	Female Optimum	0.4 mg	5.0	0.7	0.7 mg	4.9	0.1
		Male Optimum	0.3 mg	13.3	4.9	0.3 mg	20.4	4.7
Riboflavin	0.4 mg	Female Optimum	0.1 mg	4.8	5.6	0.2 mg	4.8	0.7
		Male Optimum	0.1 mg	4.2	4.7	0.0 mg	11.4	4.8
Folate	100 μg, 130 μg, 150 μg	Female Optimum	260.7 μg	5.0	0.0	336.7 μg	5.0	0.0
		Male Optimum	129.2 μg	31.7	5.0	133.6 μg	42.9	5.0
Vitamin B12	0.80 μg, 1.00 μg	Female Optimum	0.0 μg	0.5	0.0	0.0 μg	0.0	0.0
		Male Optimum	0.0 μg	0.5	0.0	0.0 μg	0.0	0.0

"Optimal Level" represents the estimated concentration of nutrient needed to achieve a post-fortification intake deficiency prevalence of 5% in a specific urban or rural area, season, and sex under maximum overage guidelines for processing, storage, and cooking (if the baseline prevalence is equal to or less than 5%, the optimal level is set to 0). For comparison, published levels are reproduced from Table 1. The projected effect of each area-, season-, and sex-specific optimal level on the prevalence of deficiency (%<EAR) is modeled for both sexes in the same area and season under maximum overage guidelines for processing, storage, and cooking. Shading indicates the extent of projected deficiency (0%: green; 50%: yellow; 100%: red).

**Table 8:** Optimal flour fortification levels for zinc, vitamin A, vitamin D, and niacin.

			**Area and Season: Rural Summer**					**Area and Season: Rural Winter**				
**Nutrient**	**Published Levels (per 100g of flour)**	**Modeled Guideline**	**Optimal Level (per 100g of flour)**	**%<EAR, Females**	**%>UL, Females**	**%<EAR, Males**	**%>UL, Males**	**Optimal Level (per 100g of flour)**	**%<EAR, Females**	**%>UL, Females**	**%<EAR, Males**	**%>UL, Males**
Iron	2.0 mg, 3.0 mg	Female Optimum	2.6 mg	5.1	0.0	0.0	2.4	1.3 mg	5.0	0.0	0.0	0.0
		Male Optimum	0.0 mg	18.7	0.0	0.5	0.0	0.0 mg	10.6	0.0	0.0	0.0
Zinc	3.0 mg, 4.0 mg	Female Optimum	0.0 mg	1.4	0.0	0.8	0.2	0.0 mg	1.9	0.0	0.5	2.5
		Male Optimum	0.0 mg	1.4	0.0	0.8	0.2	0.0 mg	1.9	0.0	0.5	2.5
Vitamin A	100 μg, 150 μg	Female Optimum	151.8 μg	5.0	0.3	12.7	5.2	267.0 μg	5.0	2.1	0.0	0.0
		Male Optimum	210.5 μg	1.0	0.3	5.0	6.0	105.5 μg	40.6	1.4	5.0	0.6
Vitamin D	55 IU	Female Optimum	209.4 IU	50.0	0.0	11.6	0.0	168.7 IU	50.0	0.0	7.6	0.0
		Male Optimum	125.7 IU	100.0	0.0	50.0	0.0	117.8 IU	89.1	0.0	50.0	0.0
Niacin	3.0 mg	Female Optimum	0.9 mg	5.0	0.0	1.8	12.9	1.1 mg	5.0	0.5	0.0	28.0
		Male Optimum	0.0 mg	11.6	0.0	3.4	6.1	0.0 mg	13.0	0.1	0.5	15.4
			**Area and Season: Urban Summer**					**Area and Season: Urban Winter**				
**Nutrient**	**Published Levels (per 100g of flour)**	**Modeled Guideline**	**Optimal Level (per 100g of flour)**	**%<EAR, Females**	**%>UL, Females**	**%<EAR, Males**	**%>UL, Males**	**Optimal Level (per 100g of flour)**	**%<EAR, Females**	**%>UL, Females**	**%<EAR, Males**	**%>UL, Males**
Iron	2.0 mg, 3.0 mg	Female Optimum	3.1 mg	5.0	0.4	0.0	0.4	4.6 mg	5.0	1.2	0.0	7.8
		Male Optimum	0.0 mg	16.8	0.0	0.2	0.0	0.0 mg	22.2	0.0	0.0	0.3
Zinc	3.0 mg, 4.0 mg	Female Optimum	0.0 mg	2.1	0.1	0.5	0.2	0.0 mg	0.3	0.0	0.8	1.0
		Male Optimum	0.0 mg	2.1	0.1	0.5	0.2	0.0 mg	0.3	0.0	0.8	1.0
Vitamin A	100 μg, 150 μg	Female Optimum	220.6 μg	5.0	1.2	6.8	5.3	395.3 μg	5.0	1.1	0.0	10.5
		Male Optimum	240.5 μg	3.8	1.3	5.0	5.7	176.0 μg	24.6	0.3	5.0	9.1
Vitamin D	55 IU	Female Optimum	178.8 IU	50.0	0.0	9.7	0.0	207.1 IU	50.0	0.0	8.0	0.0
		Male Optimum	129.4 IU	78.6	0.0	50.0	0.0	135.0 IU	85.6	0.0	50.0	0.0
Niacin	3.0 mg	Female Optimum	1.3 mg	5.0	3.4	0.5	14.6	2.0 mg	5.0	1.6	0.3	26.9
		Male Optimum	0.0 mg	10.9	1.2	2.2	6.8	0.0 mg	13.4	0.2	2.0	12.7

"Optimal Level" represents the estimated concentration of nutrient needed to achieve a post-fortification intake deficiency prevalence of 5% (50% in the case of vitamin D) in a specific urban or rural area, season, and sex under maximum overage guidelines for processing, storage, and cooking (if the baseline prevalence is equal to or less than this percentage, the optimal level is set to 0). For comparison, published are reproduced from Table 1. The projected effect of each area-, season-, and sex-specific optimal level on the prevalence of deficiency (%<EAR) and over-sufficiency (%>UL) is modeled for both sexes in the same area and season under maximum overage guidelines for processing, storage, and cooking. Shading indicates the extent of projected deficiency or over-sufficiency (0%: green; 50%: yellow; 100%: red). IU: international unit (40 IU = 1 μg).

## Discussion

This study provides seminal evidence for the expected effectiveness of mandatory industrial fortification of wheat flour, edible oil, and milk with multiple micronutrients in the Mongolian adult population. At baseline, intake deficiencies of thiamin, folate, and vitamins A, D, and E are particularly severe, with women bearing somewhat greater burdens of thiamin, vitamin A, and iron deficiency. Among different urban/rural- and sex-subgroups of the population in both summer and winter, we project that fortification of wheat flour with thiamin, folic acid, vitamin A, and vitamin D would be highly effective in reducing the prevalence of bioavailable intake deficiency of all four vitamins except vitamin D, intake of which would still increase substantially. The benefit of additionally fortifying oil with vitamins A, D, and E would be smaller but would still elicit significant increases in intake, while in the case of milk, fortification is projected to be relatively ineffective due in part to the fact that a large fraction of milk consumed in urban areas is not currently subject to industrial processing and is thus not able to be fortified. Milk fortification is still advised, however, given that this fraction is expected to shrink as Mongolia becomes increasingly commercially industrialized. Lower levels of flour fortification would be appropriate and sufficient to address intake deficiency of iron and riboflavin because baseline consumption of these micronutrients in proportion to intake requirements is relatively high in this population. This analysis alone does not support fortification of flour with zinc and niacin (projected risks of over-sufficiency outweigh reductions in deficiency), and vitamin B12 (baseline deficiency prevalence is minimal). However, fortification with these nutrients should not be discounted without considering potential benefits that may be incurred in subpopulations other than healthy men and women. In most cases, accounting for overage to compensate for losses in processing and storage, in addition to cooking losses, has a significant effect on the projected effectiveness of fortification.

### Comment on specific micronutrients

The present study demonstrated bioavailable folate intake deficiency to be extremely widespread in most of the adult population, and that fortification of wheat flour with folic acid would dramatically reduce deficiency prevalence. Prior research by Tazhibayev and colleagues demonstrated that biochemical folate deficiency could be effectively addressed in Mongolia through wheat flour fortification [15]. Folic acid fortification of wheat flour is also supported by a large body of international evidence (WHO) as to its effectiveness in preventing neural tube defects [17]. Incidentally, national statistics on these and other congenital abnormalities show a remarkably low prevalence compared to that which would be expected for Mongolia, but this is likely due to profound underestimation given inadequate enumeration of data sources and exclusion of stillbirths and terminations [38–40]. Intake deficiencies of two other B vitamins frequently added to wheat flour, thiamin and riboflavin, were found to be common in the case of thiamin (particularly among women) and relatively uncommon for riboflavin. As in most countries, information on the extent or severity of biochemical deficiency of these vitamins is unavailable for Mongolia, but it is likely that subclinical deficiency is a significant public health problem in this and other developing regions, and that their addition to wheat flour would be a prudent measure [17].

Prior research by our group and others has demonstrated extremely low levels of consumption and biochemical status of vitamin D among Mongolians of different ages across the country, a high prevalence of rickets nationwide, and that vitamin D fortification of milk consumed by school children was effective in ameliorating biochemical vitamin D deficiency [4,5,16]. We have also observed beneficial effects of vitamin D supplementation on child growth, risk of respiratory infections, and risk of atopic dermatitis in Mongolia [41–44]. Fortification models project that the range of median intake among Mongolian adults aged 22–55 after fortification of wheat flour, edible oil, and milk at levels suggested by regional and international guidelines (154.4–269.0 IU/day) exceeds median intake from food among adults aged 19–50 in the United States (144–192 IU/day), a country where most milk is fortified with vitamin D and rickets is rare despite a 94% prevalence of intake deficiency [45] (while widespread subclinical deficiency may persist at these intake levels, these levels are far above those associated with rickets and osteomalacia, which describe extreme clinical deficiency). Thus, while fortification levels modeled in the current study would not substantially reduce the prevalence of intake deficiency (defined according to a relatively conservative guideline of 400 IU/day), a potentially dramatic marginal benefit to health of increasing vitamin D intake in Mongolia may be expected given the extremely low intake at baseline [46].

Intake deficiencies of two other fat-soluble vitamins, A and E, were also found to be common in the current study and would respond well to fortification. While biochemical vitamin E status of the Mongolian population is unknown, ten percent of children aged 6 to 59 months in Mongolia are vitamin A deficient (RBP <0.7μmol/l) [5]. Furthermore, the extent of intake deficiency observed in the current study suggests that subclinical biochemical deficiency among adults may be common, which may have particular implications during pregnancy and lactation [47]. Subclinical vitamin E deficiency, while less well-characterized in terms of its consequences, may have important implications for chronic disease risk later in life [48].

An important finding of this study is the high observed baseline bioavailable intake of niacin, vitamin B12, iron, and zinc in the study population, and the correspondingly lower baseline prevalence of intake deficiency and lower marginal effectiveness of flour fortification in curbing intake deficiency of these nutrients. To the authors’ knowledge, with the exception of this study, recent data on population consumption or biochemical status of niacin, vitamin B12, or zinc are unavailable for Mongolian adults, while a moderate prevalence of biochemical iron deficiency has been observed in women but not in men [5]. National data on the prevalence of genetic iron disorders are unavailable for Mongolia. It is plausible that biochemical iron deficiency among healthy adults is at least mitigated by Mongolians’ generally high consumption of meat and organs, which are excellent sources of the more bioavailable heme form of iron and which have been implicated in high concentrations of iron found in Mongolians’ hair [49]. In fact, among 175 national food supplies, that of Mongolia was estimated to contain the fifth highest fraction of calories per capita originating from meat (18.1%) in 2013 [14], and almost all participants surveyed in this study consumed at least some meat or meat-containing product on every day of study.

### Comment on overage factors and optimal levels

This analysis also indicates the potentially severe extent to which estimated optimal levels may fail to address micronutrient intake deficiency in Mongolia if such levels are developed without appropriate application of overage factors. Model results for folate and vitamin D provide salient examples of this. Folate in flour products suffers formidable losses in processing and storage (between 10 to 60% [50]) and in baking, steaming, boiling, frying, or reheating flour or flour products (5–50% may be destroyed [51–52]). For this reason, the estimated optimal folic acid fortification level for rural females in summer of 187 μg folate/100 g wheat flour would only yield a target intake deficiency prevalence of 5% if overage is appropriately applied. If not, a projected 73.2% of this subgroup would remain deficient in summer. On the other hand, only 15–35% of vitamin D is lost in processing and storage of flour and flour products, and the vitamin is quite stable in this medium during cooking. However, given how right-skewed the distribution of vitamin D intake is in this population, even a relatively small difference in fortification levels (such as that incurred by ensuring appropriate overage) may significantly affect the projected intake deficiency prevalence. Returning to the example of rural females in summer, if overage is not applied, the estimated optimal vitamin D fortification level of 209 IU/100 g would produce a deficiency prevalence of 96.3% rather than the target prevalence of 50%.

It should be noted that in the case of folic acid, vitamin D, and other nutrients evaluated in this study, estimated optimal fortification levels for adults are generally high in comparison with reference guidelines. Such levels would not necessarily be feasible for implementation given concerns around palatability, food formulation, food safety, and cost, even in the absence of appropriate overage. These concerns are explicitly accounted for in the development of published fortification guidelines, while they are explicitly ignored in the estimation of optimal levels which are only informed by the observed distribution of nutrient intakes and nutrient requirements. Estimated optimal levels do, however, indicate the potential degree to which published guidelines may prove or fail to be effective in a given setting, as well as the direction and degree to which such guidelines should be adjusted—within the range of concentrations allowed by palatability, formulation, safety, and cost considerations—prior to their implementation. Estimated optimal levels should therefore be interpreted as a means of guidance with which to empirically tailor published guidelines to local circumstances.

We find that the range of estimated overage factors for wheat flour across seasons and subgroups in Mongolia appeared to be reasonably narrow, suggesting that while optimal fortification levels may vary significantly from one subgroup to the next, a particular overage factor will be reasonably effective in curbing processing, storage, and cooking losses across the population.

### Strengths and limitations

This study was strengthened by a rigorous baseline dietary assessment incorporating local and empirical information on recipes, dish yields, and food composition from a national sample including representation of geographic and seasonal extremes as well as urban and rural subgroups. Modeling was highly detailed and considered fortification of three vehicles separately and simultaneously, the sub-national distribution of the capacity of milk to be industrially fortified, a range of possible fortification levels suggested by national, regional, and international guidelines (and estimation of season- and subgroup-specific optimal fortification levels for comparison), estimates of fortificant stability during food processing, storage, and cooking informed by local flour consumption patterns, and estimates of nutrient bioavailability informed by food and nutrient consumption patterns. These aspects render more confidence in the accuracy of our projects as to the effectiveness of fortification, and provide a sizeable range of local guidelines to consider with respect to fortificants, food vehicles, and overage.

A limitation of this study was the need to pool data across national provinces in order to produce baseline and post-fortification estimates of nutrient intake and intake deficiency prevalence with reasonable statistical power, limiting the geographical disaggregation of our results to urban and rural areas. A second limitation was the lack of dietary data available for infants and children, pregnant and lactating women, and the elderly, making it impossible to model fortification’s effectiveness in these target groups. This must be considered in setting fortification levels, given these groups’ unique nutritional requirements and food consumption patterns. For example, while intake deficiencies of iron and zinc were found to be less common than that of other nutrients among adults in the current study, iron and zinc may be the most severely intake deficient and biochemically deficient micronutrients, respectively, among Mongolian young children [53,54]. An unmet need for nutrition research in Mongolia is periodic national surveillance of food and nutrient intake, particularly among higher-risk target groups, which should be prioritized for the purpose of updating fortification levels and informing complimentary national nutrition policies. An example of the potential importance of updating such levels is the case of industrially-produced milk, fortification of which was projected to be less effective than that of flour or oil due to its non-consumption in rural areas and low penetration in urban areas, while suggested overage for milk fortification was influenced by the fact that most milk in Mongolia is boiled as part of milk teas and milk-based soups, leading to vitamin degradation. Significant changes in the urban-rural distribution of industrial milk production, consumption, or in-home culinary practices would therefore render our models outdated. A resourceful means of dietary surveillance in Mongolia may involve application of the country’s series of frequent household consumption and expenditure surveys [34]. These surveys contain extensive information on household food consumption which may be analyzed for trends in household food vehicle or nutrient consumption, or supplemented with modules for assessing intra-household distribution of vehicles and nutrients [55].

### Conclusions for Mongolia

In conclusion, this analysis supports a policy of large-scale industrial fortification of wheat flour and wheat flour products, edible oil, and milk with iron, thiamin, riboflavin, folic acid, and vitamins A, D, and E in Mongolia. Flour fortification levels for thiamin and folate may be drawn from national guidelines published by the Mongolian University of Science and Technology, riboflavin from half the national guideline's level, and iron at the level published by the World Health Organization for countries consuming 300+ g/day of wheat flour. National guidelines for fortification of wheat flour and edible oil with vitamins A and E should be developed based on WHO and DSM international guidelines, respectively, incorporating local cost and technical considerations. National guidelines for industrial fortification of vitamins A and D in milk and vitamin D in wheat flour should also be developed by adapting guidance from existing regional standards published by the USDA and GSO, respectively. The value of zinc, niacin, and vitamin B12 fortification is not supported by this analysis for adults, however the merits of industrial fortification and alternative or simultaneous targeted strategies for delivering these nutrients should still be considered given the potentially significant health benefits for target groups not considered in this study, and weighed against the possible risks of supplying the general population with more nutrients than it may require (this also applies to thiamin, riboflavin, folic acid, and vitamins A, D, and E). While over-sufficiency is not necessarily (nor intended to be) indicative of toxicity, it bears noting that the long-term effects of potentially excessive intake of most nutrients are not well understood, and have in some cases been implicated in the increased risk of disease below or in the absence of upper limits.

Ultimately, selected fortification levels must satisfy a variety of constraints to implementation, including cultural acceptability and cost of fortification, market readiness, and capacity to implement, inspect, monitor, evaluate, and sustain a fortification program. These concerns may be addressed as appropriate by social marketing, cost effectiveness and market research, and needs assessment of the country’s relevant technical and regulatory infrastructure [17]. To ensure the effectiveness of industrial fortification in Mongolia, fortification should be mandatory, and overage for processing, storage, and cooking should be incorporated in policy guidance to accompany legislation and new or updated premix specifications. The penetration and specificity with which industrial fortification affects nutrient intake in different groups may be enhanced by considering additional potential fortification vehicles in the future. More generally, as industrial fortification is not meant to address all micronutrient intake deficiencies but rather provide a foundation for healthy nutrition, fortification itself should be accompanied by other short- and long-term supply- and demand-side approaches as part of the national nutrition strategy, including home fortification, supplementation, dietary modification, agriculture, biofortification, and economic, trade, and procurement policies geared toward diversifying the Mongolian food supply.

### Conclusions for other countries

- While WHO food fortification guidelines state that “possession of quantitative food and nutrient intake data is a prerequisite for any food fortification programme” and for projecting such a program’s impact [17], the WHO also provides recommendations on wheat and maize flour fortification based on an expert review and consensus [28]. Based on our analysis, neither estimated optimal fortification levels nor those drawn from international guidelines (including those tailored to different strata of per-capita vehicle availability) should necessarily be interpreted as superior. Modeling studies such as the one described in this paper can be useful for determining the extent to which published guidelines may benefit from adjustment prior to their application in a particular country.
- Within countries, there may be significant variation in the effectiveness of fortification across population subgroups and seasons (for example, between subgroups defined by sex, locality, and season). Such differences should be considered during the collection of baseline dietary data for setting national fortification levels, the setting of these levels themselves, and monitoring an extant fortification program.
- In setting fortification levels, it is important to incorporate overage for processing, storage, and cooking when setting fortification levels. Otherwise, the effectiveness of fortification may be diminished considerably, particularly in the case of less stable vitamins.
- Considering multiple fortification vehicles for delivery of the same and different nutrients is prudent, as it may increase the overall effectiveness of fortification and allow natural variation in population dietary patterns to be exploited for more effective targeting of intake deficiencies in different subgroups.

## Supporting information

S1 Fig Median consumption (g/day) of wheat flour, edible oil, and milk. Error bars span 95% confidence intervals estimated using 1000 bootstrap samples. Medians and associated confidence intervals were estimated using the Statistical Program to Assess Dietary Exposure (SPADE) [21]. Milk includes that which is both fortifiable (industrially-processed) and un-fortifiable (produced at home).
(DOCX)

S2 Fig Percentage contribution of consumed food groups to wheat flour and edible oil consumption. Boortsog: a deep-fried wheat-flour snack similar to a donut. Buuv: a broad category of baked wheat-flour biscuits. Huurga: a broad category of meat-based dishes made with various stir-fried and steamed ingredients. Tsuivan: a dish of steamed wheat-flour noodles and stir-fried meat. Bread foods include plain bread and bread with toppings or condiments. Dumplings include steamed, fried, and boiled dumplings.
(DOCX)

S1 Table Mean estimated overage factors for industrial fortification in Mongolia. Values represent ranges and survey-weighted means of 8 season- and subgroup-specific overage factors (factors by which fortification levels should be multiplied to compensate for losses) defined as the reciprocal of predicted nutrient losses due to processing and storage (PS) or processing, storage, and cooking (PSC). See Methods for derivation of nutrient losses and references. PSC means and ranges are omitted for iron, zinc, and B12 in flour and vitamins A and D in milk due to negligible cooking losses. PS range for iron in flour and vitamins A and D in milk are omitted due to invariant processing and storage losses observed across flour and milk products, respectively. PS and PSC ranges are omitted for vitamins A, D, and E in oil due to invariant processing, storage, and cooking losses observed across oil-containing products.
(DOCX)

S2 Table Summary of baseline and projected prevalence of vitamin and mineral intake deficiencies. Baseline and post-fortification %<EAR (estimated average requirement) represents the percentage of the population whose nutrient intake is deficient at baseline or projected to be deficient under fortification at the specified level. For vitamin D, baseline and projected median intake (in IU/day) are also provided. Statistics are weighted for the national population and projections assume fortification overage for food processing, storage, and cooking. N.S. (fortification not supported): evidence from this analysis does not support fortification of these nutrients for Mongolian adults, based on either low baseline prevalence of intake deficiency or moderate projected post-fortification prevalence of over-sufficiency. Research is warranted to determine effectiveness of fortifying these nutrients among children). IU: international unit (40 IU = 1 μg).
(DOCX)

S3 Table Prevalence of thiamin, riboflavin, folate, and vitamin B12 intake deficiency under different overage guidelines. Values represent the percentage of each subgroup’s nutrient intake lying below the subgroup-specific estimated average requirement (EAR) at baseline (Level 0) and projected under different fortification and overage guidelines. Shading indicates the extent of projected intake deficiency (0%: green; 50%: yellow; 100%: red). See Methods and Table 1 for description of levels and references. Abbreviations: PS (overage for processing and storage losses), PSC (overage for processing, storage, and cooking losses). Vitamin B12 losses in cooking flour products are negligible, therefore PSC overage for vitamin B12 is not modeled.
(DOCX)

S4 Table Prevalence of iron, zinc, vitamin E, and niacin intake deficiency and over-sufficiency under different overage guidelines. Values represent the percentage of each subgroup’s nutrient intake lying below the subgroup-specific estimated average requirement (EAR) or above its upper limit (UL), respectively, at baseline (Level 0) and projected under different fortification and overage guidelines. Shading indicates the extent of projected intake deficiency or over-sufficiency (0%: green; 50%: yellow; 100%: red). See Methods and Table 1 for description of levels and references. Abbreviations: PS (overage for processing and storage losses), PSC (overage for processing, storage, and cooking losses). Iron and zinc losses in cooking flour products are negligible, therefore PSC overage for iron and zinc is not modeled.
(DOCX)

S5 Table Prevalence of vitamins A and D intake deficiency and over-sufficiency under different overage guidelines (rural areas). Values represent the percentage of each rural subgroup’s nutrient intake lying below the subgroup-specific estimated average requirement (EAR) or above its upper limit (UL), respectively, at baseline (Level 0) and projected under different fortification and overage guidelines. Shading indicates the extent of projected intake deficiency or over-sufficiency (0%: green; 50%: yellow; 100%: red). See Methods and Table 1 for description of levels and references. Abbreviations: PS (overage for processing and storage losses), PSC (overage for processing, storage, and cooking losses).
(DOCX)

S6 Table Median intake of D under different overage guidelines (rural areas). Values represent the median intake of vitamin D (IU/day) in each rural subgroup at baseline (Level 0) and projected under different fortification and overage guidelines. Shading indicates the magnitude of projected median intake (minimum (18.2 IU/day): red; median (190.0 IU/day): yellow; estimated average requirement (400.0 IU/day): green). See Methods and Table 1 for description of levels, overage guidelines, and references, and Methods and Table 8 for description and specifications of male and female optimal levels. Abbreviations: IU (international unit; 40 IU = 1 μg), PS (overage for processing and storage losses), PSC (overage for processing, storage, and cooking losses).
(DOCX)

S7 Table Optimal flour fortification levels for thiamin, riboflavin, and folate under different overage guidelines. "Optimal Level" represents the estimated concentration of nutrient needed to achieve a post-fortification intake deficiency prevalence of 5% in a specific urban or rural area, season, and sex under maximum overage guidelines for processing, storage, and cooking (if the baseline prevalence is equal to or less than 5%, the optimal level is set to 0). For comparison, published levels are reproduced from Table 1. The projected effect of each area-, season-, and sex-specific optimal level on the prevalence of deficiency (%<EAR) is modeled for both sexes in the same area and season under different overage guidelines. Shading indicates the extent of projected deficiency (0%: green; 50%: yellow; 100%: red). Abbreviations: PS (overage for processing and storage losses), PSC (overage for processing, storage, and cooking losses). Vitamin B12 losses in cooking flour products are negligible, therefore PSC overage for vitamin B12 is not modeled.
(DOCX)

S8 Table Optimal flour fortification levels for zinc, vitamin A, vitamin D, and niacin under different overage guidelines. "Optimal Level" represents the estimated concentration of nutrient needed to achieve a post-fortification intake deficiency prevalence of 5% (50% in the case of vitamin D) in a specific urban or rural area, season, and sex under maximum overage guidelines for processing, storage, and cooking (if the baseline prevalence is equal to or less than this percentage, the optimal level is set to 0). For comparison, published are reproduced from Table 1. The projected effect of each area-, season-, and sex-specific optimal level on the prevalence of intake deficiency (%<EAR) and over-sufficiency (%>UL) is modeled for both sexes in the same area and season under different overage guidelines. Shading indicates the extent of projected deficiency or over-sufficiency (0%: green; 50%: yellow; 100%: red). Abbreviations: IU (international unit; 40 IU = 1 μg), PS (overage for processing and storage losses), PSC (overage for processing, storage, and cooking losses). Iron and zinc losses in cooking flour products are negligible, therefore PSC overage for iron and zinc is not modeled.
(DOCX)

S1 File Supplemental methods. (DOCX)

S2 File Dataset. For assistance in secondary analysis of these de-identified data, readers are welcome to contact the corresponding author (sbromage@mail.harvard.edu). Participant codes and characteristics are represented by the variables "Person-Day Code", "Sex", "Region", "Locality", "Season", "Day of Observation", "Age in Years", "Day of Week", "Month", and "Survey Weight". Nutrient intakes are represented by "Folate (ug)", "Iron (mg)", "Niacin (mg)", "Retinol (ug)", "Riboflavin (mg)", "Thiamin (mg)", "Vitamin A (ug)", "Vitamin B12 (ug)", "Vitamin D (IU)", "Vitamin E (mg)", and "Zinc (mg)". Vehicle intakes are represented by "Flour (g)", "Milk (g)", and "Oil (g)".
(XLSX)

S3 File Policy brief (English language). (DOCX)

S4 File Policy brief and summary of baseline and projected prevalence of vitamin and mineral intake deficiencies (Mongolian language). (DOCX)

S5 File External links and captions to interactive graphs of prevalence of vitamin A intake deficiency and over-sufficiency, and vitamin D intake deficiency and median intake under different overage guidelines (urban areas). (DOCX)
