# Supplementary material for: Projected effectiveness of mandatory industrial fortification of wheat flour, milk, and edible oil with multiple micronutrients among Mongolian adults
Source: PLoS One. 2018 Aug 2;13(8):e0201230. doi: 10.1371/journal.pone.0201230 (PMC6071971; doi:10.1371/journal.pone.0201230)
Supplement: S1 Table — Values represent ranges and survey-weighted means of 8 season- and subgroup-specific overage factors (factors by which fortification levels should be multiplied to compensate for losses) defined as the reciprocal of predicted nutrient losses due to processing and storage (PS) or processing, storage, and cooking (PSC). See Methods for derivation of nutrient losses and references. PSC means and ranges are omitted for iron, zinc, and B12 in flour and vitamins A and D in milk due to negligible cooking losses. PS range for iron in flour and vitamins A and D in milk are omitted due to invariant processing and storage losses observed across flour and milk products, respectively. PS and PSC ranges are omitted for vitamins A, D, and E in oil due to invariant processing, storage, and cooking losses observed across oil-containing products. (DOCX) [file pone.0201230.s003.docx]

|  |  | **Overage Guideline: PS** | | **Overage Guideline: PSC** | |
| --- | --- | --- | --- | --- | --- |
| **Vehicle** | **Nutrient** | **Mean** | **Range** | **Mean** | **Range** |
| Flour | Iron and Zinc | 1.053 |  |  |  |
|  | Vitamin A | 1.372 | 1.341-1.392 | 1.436 | 1.418-1.448 |
|  | Vitamin D | 1.279 | 1.253-1.294 | 1.279 | 1.253-1.294 |
|  | Thiamin | 1.482 | 1.426-1.508 | 1.737 | 1.638-1.787 |
|  | Riboflavin | 1.322 | 1.292-1.337 | 1.421 | 1.371-1.440 |
|  | Niacin | 1.239 | 1.220-1.249 | 1.331 | 1.306-1.344 |
|  | Folate | 1.447 | 1.383-1.486 | 1.889 | 1.841-1.910 |
|  | Vitamin B12 | 1.239 | 1.221-1.249 |  |  |
| Oil | Vitamins A and D | 1.176 |  | 1.322 |  |
|  | Vitamin E | 1.429 |  | 2.198 |  |
