# Supplementary material for: Projected effectiveness of mandatory industrial fortification of wheat flour, milk, and edible oil with multiple micronutrients among Mongolian adults
Source: PLoS One. 2018 Aug 2;13(8):e0201230. doi: 10.1371/journal.pone.0201230 (PMC6071971; doi:10.1371/journal.pone.0201230)
Supplement: S2 Table — Baseline and post-fortification %<EAR (estimated average requirement) represents the percentage of the population whose nutrient intake is deficient at baseline or projected to be deficient under fortification at the specified level. For vitamin D, baseline and projected median intake (in IU/day) are also provided. Statistics are weighted for the national population and projections assume fortification overage for food processing, storage, and cooking. N.S. (fortification not supported): evidence from this analysis does not support fortification of these nutrients for Mongolian adults, based on either low baseline prevalence of intake deficiency or moderate projected post-fortification prevalence of over-sufficiency. Research is warranted to determine effectiveness of fortifying these nutrients among children). IU: international unit (40 IU = 1 μg). (DOCX) [file pone.0201230.s004.docx]

|  |  |  |  | **Baseline %<EAR** | | **Post-Fortification %<EAR** | |
| --- | --- | --- | --- | --- | --- | --- | --- |
| **Nutrient** | **Fortificant** | **Vehicle** | **Level (per 100g)** | **Summer** | **Winter** | **Summer** | **Winter** |
| Iron | Ferrous fumarate | Flour | 2.0 mg | 9 | 10 | 4 | 5 |
| Zinc | Zinc Oxide |  | N/A | 1 | 1 | N.S. | N.S. |
| Thiamin | Thiamin mononitrate |  | 0.4 mg | 54 | 68 | 3 | 7 |
| Riboflavin | Riboflavin |  | 0.2 mg | 8 | 7 | 1 | 2 |
| Niacin | Nicotinamide |  | N/A | 7 | 8 | N.S. | N.S. |
| Folate | Folic acid |  | 115 μg | 99 | 97 | 6 | 9 |
| Vitamin B12 | Cyanocoabalamin |  | N/A | 0 | 0 | N.S. | N.S. |
| Vitamin A | Retinol palmitate | Flour | 117 μg | 53 | 59 | 9 | 17 |
|  |  | Oil | 900 μg |  |  |  |  |
|  |  | Milk | 62 μg |  |  |  |  |
| Vitamin D | Cholecalciferol | Flour | 55 IU | 100  (42 IU/day) | 100  (28 IU/day) | 97  (213 IU/day) | 99  (202 IU/day) |
|  |  | Oil | 300 IU |  |  |  |  |
|  |  | Milk | 42 IU |  |  |  |  |
| Vitamin E | Alpha tocopherol | Oil | 10.7 mg | 99 | 99 | 95 | 96 |
