# Supplementary material for: Projected effectiveness of mandatory industrial fortification of wheat flour, milk, and edible oil with multiple micronutrients among Mongolian adults
Source: PLoS One. 2018 Aug 2;13(8):e0201230. doi: 10.1371/journal.pone.0201230 (PMC6071971; doi:10.1371/journal.pone.0201230)
Supplement: S3 Table — Values represent the percentage of each subgroup’s nutrient intake lying below the subgroup-specific estimated average requirement (EAR) at baseline (Level 0) and projected under different fortification and overage guidelines. Shading indicates the extent of projected intake deficiency (0%: green; 50%: yellow; 100%: red). See Methods and Table 1 for description of levels and references. Abbreviations: PS (overage for processing and storage losses), PSC (overage for processing, storage, and cooking losses). Vitamin B12 losses in cooking flour products are negligible, therefore PSC overage for vitamin B12 is not modeled. (DOCX) [file pone.0201230.s005.docx]

|  | | | **Rural Females** | | **Rural Males** | | **Urban Females** | | **Urban Males** | |
| --- | --- | --- | --- | --- | --- | --- | --- | --- | --- | --- |
| **Nutrient** | **Fortification Level** | **Overage Guideline** | **Summer** | **Winter** | **Summer** | **Winter** | **Summer** | **Winter** | **Summer** | **Winter** |
| Thiamin | 0 | None | 65.0 | 81.9 | 36.2 | 53.4 | 58.4 | 73.5 | 53.3 | 62.5 |
|  | 1 | None | 29.7 | 43.9 | 16.1 | 9.6 | 31.8 | 48.9 | 22.3 | 31.9 |
|  |  | PS | 18.8 | 29.8 | 10.9 | 3.8 | 22.6 | 38.7 | 12.9 | 20.2 |
|  |  | PSC | 14.3 | 23.2 | 9.2 | 2.4 | 19.0 | 34.1 | 9.5 | 15.0 |
|  | 2 | None | 8.5 | 17.8 | 7.1 | 1.4 | 15.8 | 30.5 | 7.1 | 11.9 |
|  |  | PS | 2.3 | 7.5 | 3.6 | 0.2 | 8.2 | 18.8 | 1.9 | 4.0 |
|  |  | PSC | 1.2 | 4.4 | 2.6 | 0.1 | 5.8 | 14.7 | 1.0 | 2.1 |
|  | 3 | None | 1.8 | 6.8 | 3.4 | 0.2 | 8.1 | 18.8 | 1.9 | 4.0 |
|  |  | PS | 0.2 | 2.0 | 1.4 | 0.0 | 3.1 | 9.5 | 0.3 | 0.7 |
|  |  | PSC | 0.0 | 1.0 | 1.0 | 0.0 | 2.0 | 6.9 | 0.1 | 0.3 |
|  | 4 | None | 0.3 | 2.7 | 1.7 | 0.0 | 4.2 | 11.8 | 0.5 | 1.2 |
|  |  | PS | 0.0 | 0.6 | 0.6 | 0.0 | 1.3 | 5.3 | 0.0 | 0.2 |
|  |  | PSC | 0.0 | 0.3 | 0.4 | 0.0 | 0.8 | 3.5 | 0.0 | 0.0 |
| Riboflavin | 0 | None | 3.4 | 10.9 | 5.3 | 0.6 | 8.0 | 11.4 | 11.2 | 4.8 |
|  | 1 | None | 0.6 | 2.3 | 1.7 | 0.0 | 3.3 | 5.9 | 3.4 | 1.3 |
|  |  | PS | 0.4 | 1.4 | 1.3 | 0.0 | 2.4 | 4.9 | 2.4 | 0.8 |
|  |  | PSC | 0.3 | 1.3 | 1.2 | 0.0 | 2.3 | 4.6 | 2.1 | 0.6 |
|  | 2 | None | 0.1 | 0.5 | 0.8 | 0.0 | 1.4 | 3.3 | 1.0 | 0.2 |
|  |  | PS | 0.0 | 0.2 | 0.5 | 0.0 | 0.9 | 2.0 | 0.5 | 0.0 |
|  |  | PSC | 0.0 | 0.2 | 0.5 | 0.0 | 0.8 | 1.8 | 0.5 | 0.0 |
|  | 3 | None | 0.0 | 0.1 | 0.4 | 0.0 | 0.7 | 1.7 | 0.4 | 0.0 |
|  |  | PS | 0.0 | 0.0 | 0.3 | 0.0 | 0.4 | 1.0 | 0.1 | 0.0 |
|  |  | PSC | 0.0 | 0.0 | 0.2 | 0.0 | 0.3 | 0.9 | 0.1 | 0.0 |
|  | 4 | None | 0.0 | 0.0 | 0.3 | 0.0 | 0.4 | 1.0 | 0.1 | 0.0 |
|  |  | PS | 0.0 | 0.0 | 0.2 | 0.0 | 0.2 | 0.6 | 0.0 | 0.0 |
|  |  | PSC | 0.0 | 0.0 | 0.2 | 0.0 | 0.1 | 0.5 | 0.0 | 0.0 |
| Folate | 0 | None | 100.0 | 99.3 | 97.1 | 97.1 | 99.7 | 99.0 | 98.0 | 94.4 |
|  | 1 | None | 83.1 | 54.4 | 31.3 | 11.0 | 55.4 | 67.8 | 24.7 | 26.1 |
|  |  | PS | 41.0 | 29.7 | 13.9 | 2.2 | 30.9 | 46.0 | 5.0 | 6.5 |
|  |  | PSC | 12.3 | 12.4 | 6.3 | 0.3 | 18.7 | 29.4 | 1.0 | 1.4 |
|  | 2 | None | 70.6 | 43.6 | 24.0 | 5.9 | 46.6 | 60.3 | 15.3 | 16.3 |
|  |  | PS | 22.3 | 20.4 | 9.4 | 1.0 | 23.4 | 37.2 | 2.2 | 3.5 |
|  |  | PSC | 3.9 | 7.4 | 4.3 | 0.1 | 13.1 | 22.1 | 0.3 | 0.6 |
|  | 3 | None | 55.5 | 34.2 | 18.2 | 3.1 | 38.9 | 52.9 | 9.2 | 10.6 |
|  |  | PS | 10.9 | 14.3 | 6.4 | 0.4 | 17.8 | 29.9 | 1.0 | 1.6 |
|  |  | PSC | 1.2 | 4.4 | 2.8 | 0.0 | 9.3 | 16.8 | 0.1 | 0.3 |
|  | 4 | None | 35.5 | 24.2 | 12.8 | 1.3 | 30.4 | 44.0 | 4.5 | 5.5 |
|  |  | PS | 3.3 | 8.5 | 4.0 | 0.2 | 12.2 | 22.4 | 0.3 | 0.6 |
|  |  | PSC | 0.2 | 2.4 | 1.5 | 0.0 | 5.8 | 11.6 | 0.0 | 0.1 |
| Vitamin B12 | 0 | None | 0.0 | 0.0 | 0.0 | 0.0 | 0.5 | 0.0 | 0.0 | 0.0 |
|  | 1 | None | 0.0 | 0.0 | 0.0 | 0.0 | 0.0 | 0.0 | 0.0 | 0.0 |
|  |  | PS | 0.0 | 0.0 | 0.0 | 0.0 | 0.0 | 0.0 | 0.0 | 0.0 |
|  | 2 | None | 0.0 | 0.0 | 0.0 | 0.0 | 0.0 | 0.0 | 0.0 | 0.0 |
|  |  | PS | 0.0 | 0.0 | 0.0 | 0.0 | 0.0 | 0.0 | 0.0 | 0.0 |
|  | 3 | None | 0.0 | 0.0 | 0.0 | 0.0 | 0.0 | 0.0 | 0.0 | 0.0 |
|  |  | PS | 0.0 | 0.0 | 0.0 | 0.0 | 0.0 | 0.0 | 0.0 | 0.0 |
|  | 4 | None | 0.0 | 0.0 | 0.0 | 0.0 | 0.0 | 0.0 | 0.0 | 0.0 |
|  |  | PS | 0.0 | 0.0 | 0.0 | 0.0 | 0.0 | 0.0 | 0.0 | 0.0 |
