# Supplementary material for: Projected effectiveness of mandatory industrial fortification of wheat flour, milk, and edible oil with multiple micronutrients among Mongolian adults
Source: PLoS One. 2018 Aug 2;13(8):e0201230. doi: 10.1371/journal.pone.0201230 (PMC6071971; doi:10.1371/journal.pone.0201230)
Supplement: S5 Table — Values represent the percentage of each rural subgroup’s nutrient intake lying below the subgroup-specific estimated average requirement (EAR) or above its upper limit (UL), respectively, at baseline (Level 0) and projected under different fortification and overage guidelines. Shading indicates the extent of projected intake deficiency or over-sufficiency (0%: green; 50%: yellow; 100%: red). See Methods and Table 1 for description of levels and references. Abbreviations: PS (overage for processing and storage losses), PSC (overage for processing, storage, and cooking losses). (DOCX) [file pone.0201230.s007.docx]

|  | | | **Vitamin A** | | | | | | | | **Vitamin D** | | | | | | | |
| --- | --- | --- | --- | --- | --- | --- | --- | --- | --- | --- | --- | --- | --- | --- | --- | --- | --- | --- |
|  | | | **Females** | | | | **Males** | | | | **Females** | | | | **Males** | | | |
|  | | | **Summer** | | **Winter** | | **Summer** | | **Winter** | | **Summer** | | **Winter** | | **Summer** | | **Winter** | |
|  | | | **%< EAR** | **%> UL** | **%< EAR** | **%> UL** | **%< EAR** | **%> UL** | **%< EAR** | **%> UL** | **%< EAR** | **%> UL** | **%< EAR** | **%> UL** | **%< EAR** | **%> UL** | **%< EAR** | **%> UL** |
| 0 | 0 | None | 40 | 0.6 | 71.3 | 0.4 | 49.1 | 2.8 | 41.5 | 5.2 | 100 | 0 | 100 | 0 | 100 | 0 | 100 | 0 |
|  | 1 | None | 38.1 | 0.7 | 68.5 | 0.2 | 46.8 | 5.5 | 36.4 | 6.8 | 100 | 0 | 100 | 0 | 100 | 0 | 100 | 0 |
|  |  | PS | 37.3 | 0.7 | 68.1 | 0.1 | 45.8 | 5.4 | 35 | 6.8 | 100 | 0 | 100 | 0 | 100 | 0 | 100 | 0 |
|  |  | PSC | 35.8 | 2.1 | 67.3 | 0 | 43.5 | 5.3 | 32.7 | 6.3 | 100 | 0 | 100 | 0 | 100 | 0 | 100 | 0 |
|  | 2 | None | 35.8 | 2.1 | 67.5 | 0.1 | 44.1 | 5.3 | 33.2 | 6.4 | 100 | 0 | 100 | 0 | 100 | 0 | 100 | 0 |
|  |  | PS | 34.4 | 1.8 | 65.8 | 0 | 41.2 | 5.3 | 31.4 | 2.9 | 100 | 0 | 100 | 0 | 100 | 0 | 100 | 0 |
|  |  | PSC | 30.5 | 1.3 | 61.2 | 0 | 35.6 | 5.1 | 26.9 | 2.2 | 100 | 0 | 100 | 0 | 100 | 0 | 100 | 0 |
|  | 3 | None | 34.3 | 1.7 | 65.6 | 0 | 40.7 | 5.3 | 31 | 2.8 | 100 | 0 | 100 | 0 | 100 | 0 | 100 | 0 |
|  |  | PS | 31.2 | 1.4 | 61.5 | 0 | 35.9 | 5.1 | 27.1 | 2.2 | 100 | 0 | 100 | 0 | 100 | 0 | 100 | 0 |
|  |  | PSC | 23.9 | 0.8 | 54.5 | 0 | 27.9 | 5 | 20.5 | 1.2 | 100 | 0 | 100 | 0 | 100 | 0 | 100 | 0 |
|  | 4 | None | 31.6 | 1.4 | 62.3 | 0 | 36.9 | 5.2 | 28 | 2.4 | 100 | 0 | 100 | 0 | 100 | 0 | 100 | 0 |
|  |  | PS | 26.1 | 1.1 | 57.1 | 0 | 30.9 | 5.1 | 22.9 | 1.6 | 100 | 0 | 100 | 0 | 100 | 0 | 100 | 0 |
|  |  | PSC | 18.2 | 0.6 | 44.7 | 0 | 21.1 | 5.1 | 15 | 0.7 | 100 | 0 | 100 | 0 | 100 | 0 | 100 | 0 |
| 1 | 0 | None | 25.2 | 1.8 | 53.6 | 1.2 | 33.7 | 4.3 | 18.5 | 2.4 | 100 | 0 | 100 | 0 | 100 | 0 | 100 | 0 |
|  |  | PS | 18.2 | 1.6 | 47.2 | 1.3 | 27.2 | 4.5 | 10.6 | 1.1 | 100 | 0 | 100 | 0 | 99.8 | 0 | 100 | 0 |
|  |  | PSC | 17 | 1.6 | 45.5 | 1.4 | 26.4 | 4.5 | 9.1 | 0.9 | 100 | 0 | 100 | 0 | 99.8 | 0 | 100 | 0 |
|  | 1 | None | 22.6 | 1.5 | 50 | 1 | 30.9 | 4.6 | 13.9 | 1.9 | 100 | 0 | 100 | 0 | 99.9 | 0 | 100 | 0 |
|  |  | PS | 15.3 | 1.3 | 41.8 | 1.1 | 23.1 | 4.9 | 6.7 | 0.8 | 100 | 0 | 100 | 0 | 99.7 | 0 | 100 | 0 |
|  |  | PSC | 13.1 | 1.1 | 37.4 | 1 | 19.6 | 5 | 3.9 | 0.5 | 100 | 0 | 100 | 0 | 99.5 | 0 | 100 | 0 |
|  | 2 | None | 20.9 | 1.3 | 47.1 | 0.9 | 27.6 | 4.6 | 10.9 | 1.4 | 100 | 0 | 100 | 0 | 99.8 | 0 | 100 | 0 |
|  |  | PS | 12.7 | 1 | 36.9 | 0.9 | 18.6 | 5 | 3.9 | 0.5 | 100 | 0 | 100 | 0 | 99.4 | 0 | 100 | 0 |
|  |  | PSC | 9.5 | 0.8 | 29.9 | 0.7 | 14 | 5.2 | 1.5 | 0.2 | 100 | 0 | 100 | 0 | 98.9 | 0 | 100 | 0 |
|  | 3 | None | 18.6 | 1.2 | 44.1 | 0.7 | 24.3 | 4.6 | 8.5 | 1 | 100 | 0 | 100 | 0 | 99.7 | 0 | 100 | 0 |
|  |  | PS | 10.4 | 0.9 | 32.1 | 0.7 | 15.1 | 5.1 | 2.1 | 0.3 | 100 | 0 | 100 | 0 | 99 | 0 | 100 | 0 |
|  |  | PSC | 6.5 | 0.7 | 22.8 | 0.6 | 9.5 | 5.3 | 0.4 | 0.2 | 100 | 0 | 100 | 0 | 97.5 | 0 | 100 | 0 |
|  | 4 | None | 16.7 | 1.1 | 40.9 | 0.6 | 21 | 4.7 | 6.3 | 0.8 | 100 | 0 | 100 | 0 | 99.6 | 0 | 100 | 0 |
|  |  | PS | 8.1 | 0.7 | 27.2 | 0.6 | 11.7 | 5.2 | 1 | 0.2 | 100 | 0 | 100 | 0 | 98.2 | 0 | 100 | 0 |
|  |  | PSC | 4.3 | 0.4 | 15.8 | 0.5 | 6.5 | 5.5 | 0.1 | 0 | 100 | 0 | 99.9 | 0 | 95.1 | 0 | 99.5 | 0 |
| 2 | 0 | None | 22.1 | 1.8 | 50.7 | 1.2 | 30.9 | 4.4 | 14.1 | 1.7 | 100 | 0 | 100 | 0 | 98.6 | 0 | 100 | 0 |
|  |  | PS | 14.3 | 1.5 | 42.6 | 1.4 | 23.6 | 4.6 | 6.6 | 0.7 | 100 | 0 | 100 | 0 | 96.5 | 0 | 100 | 0 |
|  |  | PSC | 13.3 | 1.5 | 40.8 | 1.4 | 22.5 | 4.6 | 5.2 | 0.6 | 100 | 0 | 100 | 0 | 96.5 | 0 | 100 | 0 |
|  | 1 | None | 20 | 1.4 | 46.8 | 1.2 | 28.1 | 4.6 | 10.8 | 1.3 | 100 | 0 | 100 | 0 | 98.4 | 0 | 100 | 0 |
|  |  | PS | 12.2 | 1.2 | 36.8 | 1.2 | 19.2 | 4.9 | 3.8 | 0.5 | 100 | 0 | 100 | 0 | 94.9 | 0 | 100 | 0 |
|  |  | PSC | 10.1 | 0.9 | 32.6 | 1.2 | 16.4 | 5.2 | 1.9 | 0.3 | 100 | 0 | 100 | 0 | 93.8 | 0 | 100 | 0 |
|  | 2 | None | 17.6 | 1.3 | 43.5 | 1 | 24.7 | 4.8 | 8.1 | 1 | 100 | 0 | 100 | 0 | 97.8 | 0 | 100 | 0 |
|  |  | PS | 9.9 | 0.9 | 32.1 | 1 | 15.7 | 5.2 | 1.9 | 0.3 | 100 | 0 | 100 | 0 | 92.9 | 0 | 99.8 | 0 |
|  |  | PSC | 7 | 0.8 | 24.9 | 0.8 | 11.2 | 5.3 | 0.6 | 0.1 | 100 | 0 | 99.9 | 0 | 89.7 | 0 | 99.1 | 0 |
|  | 3 | None | 16.1 | 1.1 | 40.3 | 0.8 | 21.8 | 4.8 | 5.9 | 0.7 | 100 | 0 | 100 | 0 | 96.9 | 0 | 100 | 0 |
|  |  | PS | 7.8 | 0.8 | 27.3 | 0.8 | 12.4 | 5.3 | 0.9 | 0.2 | 100 | 0 | 99.9 | 0 | 90 | 0 | 99.1 | 0 |
|  |  | PSC | 4.9 | 0.6 | 19.1 | 0.7 | 7.6 | 5.6 | 0.1 | 0 | 100 | 0 | 99.6 | 0 | 84.6 | 0 | 95.6 | 0 |
|  | 4 | None | 13.6 | 1 | 37 | 0.7 | 18.4 | 4.9 | 3.9 | 0.5 | 100 | 0 | 100 | 0 | 95.9 | 0 | 100 | 0 |
|  |  | PS | 6.3 | 0.7 | 23.2 | 0.7 | 9.6 | 5.4 | 0.4 | 0.1 | 100 | 0 | 99.7 | 0 | 86.9 | 0 | 97.7 | 0 |
|  |  | PSC | 3.2 | 0.3 | 13.6 | 0.6 | 5.1 | 5.8 | 0 | 0 | 100 | 0 | 98.5 | 0 | 78.5 | 0 | 88.8 | 0 |
| 3 | 0 | None | 19 | 1.6 | 47.4 | 1.3 | 27.9 | 4.5 | 10.8 | 1.2 | 100 | 0 | 100 | 0 | 93.6 | 0 | 100 | 0 |
|  |  | PS | 11.1 | 1.3 | 37.9 | 1.5 | 20.1 | 4.7 | 3.4 | 0.4 | 100 | 0 | 99.6 | 0 | 83.1 | 0 | 96.5 | 0 |
|  |  | PSC | 10.2 | 1.3 | 35.6 | 1.5 | 19 | 4.8 | 2.5 | 0.3 | 100 | 0 | 99.6 | 0 | 83.1 | 0 | 96.5 | 0 |
|  | 1 | None | 17.2 | 1.3 | 43.6 | 1.2 | 25.2 | 4.7 | 7.9 | 0.9 | 100 | 0 | 100 | 0 | 91.9 | 0 | 99.7 | 0 |
|  |  | PS | 9.4 | 1 | 32.3 | 1.3 | 16 | 5.1 | 1.9 | 0.3 | 100 | 0 | 99.1 | 0 | 79.2 | 0 | 92.7 | 0 |
|  |  | PSC | 7.4 | 0.9 | 27.9 | 1.2 | 13.7 | 5.4 | 0.8 | 0.2 | 100 | 0 | 98.7 | 0 | 77.1 | 0 | 89.8 | 0 |
|  | 2 | None | 15 | 1.2 | 39.9 | 1 | 21.5 | 4.9 | 5.6 | 0.7 | 100 | 0 | 99.9 | 0 | 90 | 0 | 99.2 | 0 |
|  |  | PS | 7.5 | 0.8 | 27.6 | 1.1 | 13.2 | 5.4 | 0.8 | 0.1 | 100 | 0 | 98.4 | 0 | 75.2 | 0 | 87.3 | 0 |
|  |  | PSC | 4.7 | 0.7 | 21.1 | 0.9 | 9.2 | 5.5 | 0.2 | 0 | 100 | 0 | 97 | 0 | 70.3 | 0 | 79.1 | 0 |
|  | 3 | None | 13.8 | 1.1 | 36.8 | 0.9 | 18.8 | 5 | 3.8 | 0.5 | 100 | 0 | 99.8 | 0 | 88.1 | 0 | 98 | 0 |
|  |  | PS | 5.5 | 0.7 | 22.9 | 0.9 | 10.1 | 5.5 | 0.3 | 0 | 100 | 0 | 97.1 | 0 | 70.7 | 0 | 79.7 | 0 |
|  |  | PSC | 3.3 | 0.6 | 15.6 | 0.8 | 6.2 | 6 | 0 | 0 | 100 | 0 | 94.5 | 0 | 63.1 | 0 | 67.5 | 0 |
|  | 4 | None | 11.6 | 0.9 | 33.2 | 0.7 | 16.2 | 5.1 | 2.4 | 0.3 | 100 | 0 | 99.6 | 0 | 85.4 | 0 | 96.3 | 0 |
|  |  | PS | 4.5 | 0.6 | 19 | 0.8 | 7.7 | 5.7 | 0.1 | 0 | 100 | 0 | 95.7 | 0 | 66 | 0 | 72.6 | 0 |
|  |  | PSC | 2 | 0.3 | 10.7 | 0.6 | 4.3 | 6.2 | 0 | 0 | 99.8 | 0 | 90.8 | 0 | 55.2 | 0 | 55.9 | 0 |
| 4 | 0 | None | 16.2 | 1.6 | 44 | 1.4 | 25.4 | 4.5 | 7.7 | 0.8 | 100 | 0 | 99.1 | 0 | 81.1 | 0 | 93.1 | 0 |
|  |  | PS | 8.4 | 1.3 | 33.6 | 1.5 | 17.1 | 4.9 | 1.6 | 0.2 | 100 | 0 | 93.3 | 0 | 61 | 0 | 63.2 | 0 |
|  |  | PSC | 7.6 | 0.3 | 31.1 | 1.5 | 15.9 | 5 | 1 | 0.2 | 100 | 0 | 93.3 | 0 | 61 | 0 | 63.2 | 0 |
|  | 1 | None | 14.4 | 1.3 | 39.8 | 1.2 | 22.1 | 4.9 | 5.2 | 0.6 | 100 | 0 | 98.6 | 0 | 78.2 | 0 | 89.7 | 0 |
|  |  | PS | 7 | 0.9 | 28 | 1.3 | 13.7 | 5.3 | 0.8 | 0.1 | 100 | 0 | 90.6 | 0 | 57 | 0 | 55.1 | 0 |
|  |  | PSC | 5.1 | 0.7 | 23.3 | 1.2 | 11.3 | 5.5 | 0.2 | 0 | 100 | 0 | 89.2 | 0 | 54.6 | 0 | 51 | 0 |
|  | 2 | None | 12.8 | 1.1 | 36.2 | 1.1 | 19.3 | 5 | 3.4 | 0.5 | 100 | 0 | 98 | 0 | 75.2 | 0 | 85.1 | 0 |
|  |  | PS | 5.3 | 0.7 | 23.3 | 1.1 | 10.4 | 5.5 | 0.2 | 0 | 100 | 0 | 88 | 0 | 52.5 | 0 | 47.5 | 0 |
|  |  | PSC | 3.4 | 0.6 | 17.8 | 1 | 7.5 | 5.9 | 0 | 0 | 99.8 | 0 | 84.8 | 0 | 47.9 | 0 | 40.3 | 0 |
|  | 3 | None | 11 | 1 | 33.1 | 1 | 16.6 | 5.1 | 2.2 | 0.3 | 100 | 0 | 97.2 | 0 | 72.3 | 0 | 80.2 | 0 |
|  |  | PS | 4 | 0.6 | 19.8 | 1 | 8.3 | 5.8 | 0.1 | 0 | 99.8 | 0 | 85.1 | 0 | 48.2 | 0 | 40.8 | 0 |
|  |  | PSC | 2.2 | 0.5 | 12.4 | 0.9 | 5 | 6.2 | 0 | 0 | 99.1 | 0 | 79 | 0 | 41.3 | 0 | 31.1 | 0 |
|  | 4 | None | 9.6 | 0.8 | 29.7 | 0.8 | 14.2 | 5.2 | 1.4 | 0.2 | 100 | 0 | 96 | 0 | 69.1 | 0 | 74.3 | 0 |
|  |  | PS | 3.1 | 0.6 | 16 | 0.8 | 6.3 | 6 | 0 | 0 | 99.4 | 0 | 81.2 | 0 | 44.2 | 0 | 35.2 | 0 |
|  |  | PSC | 1.3 | 0.3 | 8.4 | 0.8 | 3.4 | 6.6 | 0 | 0 | 97.7 | 0 | 73.6 | 0 | 35.1 | 0 | 24 | 0 |
