# Supplementary material for: Projected effectiveness of mandatory industrial fortification of wheat flour, milk, and edible oil with multiple micronutrients among Mongolian adults
Source: PLoS One. 2018 Aug 2;13(8):e0201230. doi: 10.1371/journal.pone.0201230 (PMC6071971; doi:10.1371/journal.pone.0201230)
Supplement: S6 Table — Values represent the median intake of vitamin D (IU/day) in each rural subgroup at baseline (Level 0) and projected under different fortification and overage guidelines. Shading indicates the magnitude of projected median intake (minimum (18.2 IU/day): red; median (190.0 IU/day): yellow; estimated average requirement (400.0 IU/day): green). See Methods and Table 1 for description of levels, overage guidelines, and references, and Methods and Table 8 for description and specifications of male and female optimal levels. Abbreviations: IU (international unit; 40 IU = 1 μg), PS (overage for processing and storage losses), PSC (overage for processing, storage, and cooking losses). (DOCX) [file pone.0201230.s008.docx]

|  | | **Females** | | | | | | **Males** | | | | | |
| --- | --- | --- | --- | --- | --- | --- | --- | --- | --- | --- | --- | --- | --- |
|  | | **Summer** | | | **Winter** | | | **Summer** | | | **Winter** | | |
| **Flour Fortification Level** | **Oil Fortification Level** | **No Overage** | **PS Overage** | **PSC Overage** | **No Overage** | **PS Overage** | **PSC Overage** | **No Overage** | **PS Overage** | **PSC Overage** | **No Overage** | **PS Overage** | **PSC Overage** |
| 0 | 0 | 29.1 | 29.1 | 29.1 | 18.2 | 18.2 | 18.2 | 41.1 | 41.1 | 41.1 | 31.3 | 31.3 | 31.3 |
|  | 1 | 34.5 | 36.6 | 40.8 | 27.4 | 30.9 | 36.8 | 54.1 | 59.9 | 70.0 | 44.3 | 49.4 | 58.1 |
|  | 2 | 39.7 | 44.3 | 52.1 | 35.3 | 41.9 | 53.2 | 67.6 | 78.4 | 97.2 | 56.0 | 65.6 | 82.8 |
|  | 3 | 45.1 | 51.6 | 63.0 | 43.0 | 52.3 | 69.5 | 80.1 | 95.8 | 122.9 | 67.2 | 81.4 | 107.1 |
|  | 4 | 50.1 | 58.6 | 73.8 | 50.3 | 62.9 | 85.3 | 92.3 | 112.6 | 148.7 | 78.4 | 97.3 | 131.6 |
| 1 | 0 | 67.8 | 78.5 | 78.5 | 69.4 | 82.0 | 82.0 | 105.5 | 123.4 | 123.4 | 102.1 | 119.5 | 119.5 |
|  | 1 | 72.8 | 85.8 | 89.8 | 76.9 | 92.7 | 98.2 | 117.4 | 140.0 | 148.7 | 113.3 | 135.3 | 143.9 |
|  | 2 | 78.3 | 93.1 | 100.8 | 84.3 | 103.1 | 114.4 | 128.9 | 156.6 | 174.7 | 124.3 | 151.1 | 167.7 |
|  | 3 | 83.4 | 100.2 | 111.8 | 91.7 | 113.6 | 130.3 | 140.9 | 173.4 | 200.1 | 135.6 | 166.6 | 191.9 |
|  | 4 | 88.4 | 107.4 | 122.6 | 99.0 | 123.9 | 146.3 | 152.8 | 189.7 | 225.7 | 146.3 | 182.3 | 215.8 |
| 2 | 0 | 105.7 | 127.3 | 127.3 | 119.0 | 144.0 | 144.0 | 167.7 | 202.2 | 202.2 | 170.3 | 204.6 | 204.6 |
|  | 1 | 110.7 | 134.4 | 138.2 | 126.5 | 154.6 | 160.3 | 178.8 | 218.4 | 228.0 | 181.3 | 220.3 | 228.8 |
|  | 2 | 115.8 | 141.5 | 149.3 | 133.8 | 165.1 | 176.2 | 190.0 | 235.6 | 253.4 | 192.4 | 236.0 | 252.7 |
|  | 3 | 120.9 | 148.7 | 160.0 | 141.1 | 175.4 | 192.0 | 201.7 | 252.2 | 278.2 | 203.1 | 251.4 | 276.5 |
|  | 4 | 125.8 | 155.8 | 171.2 | 148.3 | 185.7 | 207.8 | 213.4 | 268.5 | 303.8 | 214.1 | 267.1 | 300.3 |
| 3 | 0 | 143.7 | 175.9 | 175.9 | 168.4 | 206.1 | 206.1 | 229.0 | 281.8 | 281.8 | 237.9 | 289.5 | 289.5 |
|  | 1 | 148.6 | 183.0 | 186.8 | 175.9 | 216.5 | 222.1 | 240.5 | 298.0 | 305.8 | 248.9 | 305.0 | 313.5 |
|  | 2 | 153.7 | 190.3 | 197.8 | 183.1 | 227.0 | 238.1 | 251.7 | 313.2 | 330.3 | 259.9 | 320.6 | 337.2 |
|  | 3 | 158.6 | 197.2 | 208.7 | 190.4 | 237.3 | 253.8 | 262.6 | 329.3 | 355.1 | 270.9 | 336.1 | 362.9 |
|  | 4 | 163.6 | 204.4 | 219.7 | 197.7 | 247.6 | 269.0 | 274.3 | 345.0 | 381.7 | 281.7 | 351.4 | 386.7 |
| 4 | 0 | 181.4 | 224.3 | 224.3 | 217.9 | 268.0 | 268.0 | 290.5 | 360.2 | 360.2 | 305.7 | 374.2 | 374.2 |
|  | 1 | 186.5 | 231.6 | 235.4 | 225.2 | 278.3 | 284.0 | 301.8 | 374.7 | 383.1 | 316.5 | 389.8 | 398.0 |
|  | 2 | 191.5 | 238.7 | 246.3 | 232.6 | 288.9 | 300.3 | 312.6 | 390.8 | 407.7 | 327.5 | 405.2 | 421.9 |
|  | 3 | 196.5 | 245.7 | 257.3 | 239.9 | 299.5 | 300.3 | 323.4 | 406.6 | 433.4 | 338.3 | 420.5 | 447.7 |
|  | 4 | 201.5 | 252.9 | 268.2 | 247.0 | 309.5 | 300.3 | 334.5 | 422.0 | 459.9 | 349.1 | 436.1 | 472.1 |
| Female Optimum | 0 | 318.3 | 400.0 | 400.0 | 323.2 | 400.0 | 400.0 | 504.1 | 635.0 | 635.0 | 452.0 | 557.9 | 557.9 |
| Male Optimum | 0 | 203.1 | 252.1 | 252.1 | 232.0 | 285.5 | 285.5 | 325.0 | 400.0 | 400.0 | 324.9 | 400.0 | 400.0 |
