# Supplementary material for: Projected effectiveness of mandatory industrial fortification of wheat flour, milk, and edible oil with multiple micronutrients among Mongolian adults
Source: PLoS One. 2018 Aug 2;13(8):e0201230. doi: 10.1371/journal.pone.0201230 (PMC6071971; doi:10.1371/journal.pone.0201230)
Supplement: S7 Table — "Optimal Level" represents the estimated concentration of nutrient needed to achieve a post-fortification intake deficiency prevalence of 5% in a specific urban or rural area, season, and sex under maximum overage guidelines for processing, storage, and cooking (if the baseline prevalence is equal to or less than 5%, the optimal level is set to 0). For comparison, published levels are reproduced from Table 1. The projected effect of each area-, season-, and sex-specific optimal level on the prevalence of deficiency (%<EAR) is modeled for both sexes in the same area and season under different overage guidelines. Shading indicates the extent of projected deficiency (0%: green; 50%: yellow; 100%: red). Abbreviations: PS (overage for processing and storage losses), PSC (overage for processing, storage, and cooking losses). Vitamin B12 losses in cooking flour products are negligible, therefore PSC overage for vitamin B12 is not modeled. (DOCX) [file pone.0201230.s009.docx]

|  | | | | **Area and Season: Rural Summer** | | | **Area and Season: Rural Winter** | | | | |
| --- | --- | --- | --- | --- | --- | --- | --- | --- | --- | --- | --- |
| **Nutrient** | **Published Levels (per 100g of flour)** | **Modeled Optimum** | **Overage Guideline** | **Optimal Level (per 100g of vehicle)** | **%<EAR, Females** | **%<EAR, Males** | **Optimal Level (per 100g of flour)** | **%<EAR, Females** | | **%<EAR, Males** | |
| Thiamine | 0.4 mg | Female Optimum | None | 0.3 mg | 18.3 | 10.9 | 0.4 mg | 18.7 | | 1.5 | |
|  |  |  | PS |  | 7.8 | 6.6 |  | 7.9 | | 0.3 | |
|  |  |  | PSC |  | 4.8 | 5.2 |  | 4.8 | | 0.1 | |
|  |  | Male Optimum | None | 0.3 mg | 17.2 | 10.5 | 0.2 mg | 51.4 | | 14.1 | |
|  |  |  | PS |  | 7.0 | 6.2 |  | 38.3 | | 6.9 | |
|  |  |  | PSC |  | 4.3 | 4.9 |  | 31.8 | | 4.4 | |
| Riboflavin | 0.4 mg | Female Optimum | None | 0.0 mg | 3.4 | 5.3 | 0.1 mg | 6.1 | | 0.2 | |
|  |  |  | PS |  | 3.4 | 5.3 |  | 5.0 | | 0.1 | |
|  |  |  | PSC |  | 3.4 | 5.3 |  | 4.7 | | 0.0 | |
|  |  | Male Optimum | None | 0.0 mg | 3.1 | 5.0 | 0.0 mg | 10.9 | | 0.6 | |
|  |  |  | PS |  | 3.1 | 4.9 |  | 10.9 | | 0.6 | |
|  |  |  | PSC |  | 3.1 | 4.9 |  | 10.9 | | 0.6 | |
| Folate | 100 μg, 130 μg,  150 μg | Female Optimum | None | 187.0 μg | 73.2 | 25.2 | 210.7 μg | 36.4 | | 3.6 | |
|  |  |  | PS |  | 25.4 | 10.2 |  | 15.6 | | 0.6 | |
|  |  |  | PSC |  | 4.9 | 4.7 |  | 5.0 | | 0.0 | |
|  |  | Male Optimum | None | 183.2 μg | 75.3 | 26.3 | 104.1 μg | 82.7 | | 42.3 | |
|  |  |  | PS |  | 28.0 | 10.8 |  | 65.0 | | 18.9 | |
|  |  |  | PSC |  | 6.1 | 5.0 |  | 42.1 | | 5.0 | |
| Vitamin B12 | 0.80 μg, 1.00 μg | Female Optimum | None | 0.0 μg | 0.0 | 0.0 | 0.0 μg | 0.0 | | 0.0 | |
|  |  |  | PS |  | 0.0 | 0.0 |  | 0.0 | | 0.0 | |
|  |  | Male Optimum | None | 0.0 μg | 0.0 | 0.0 | 0.0 μg | 0.0 | | 0.0 | |
|  |  |  | PS |  | 0.0 | 0.0 |  | 0.0 | | 0.0 | |
|  | | | | **Area and Season: Urban Summer** | | | **Area and Season: Urban Winter** | | | | |
| **Nutrient** | **Published Levels (per 100g of flour)** | **Modeled Optimum** | **Overage Guideline** | **Optimal Level (per 100g of vehicle)** | **%<EAR, Females** | **%<EAR, Males** | **Optimal Level (per 100g of flour)** | **%<EAR, Females** | **%<EAR, Males** | |  |
| Thiamine | 0.4 mg | Female Optimum | None | 0.4 mg | 14.3 | 5.9 | 0.7 mg | 14.9 | 2.2 | |  |
|  |  |  | PS |  | 6.9 | 1.4 |  | 7.0 | 0.3 | |  |
|  |  |  | PSC |  | 5.0 | 0.7 |  | 4.9 | 0.1 | |  |
|  |  | Male Optimum | None | 0.3 mg | 25.9 | 15.8 | 0.3 mg | 36.8 | 18.3 | |  |
|  |  |  | PS |  | 16.4 | 7.5 |  | 25.1 | 7.6 | |  |
|  |  |  | PSC |  | 13.3 | 4.9 |  | 20.4 | 4.7 | |  |
| Riboflavin | 0.4 mg | Female Optimum | None | 0.1 mg | 5.5 | 6.9 | 0.2 mg | 6.1 | 1.4 | |  |
|  |  |  | PS |  | 5.0 | 5.9 |  | 5.1 | 0.9 | |  |
|  |  |  | PSC |  | 4.8 | 5.6 |  | 4.8 | 0.7 | |  |
|  |  | Male Optimum | None | 0.1 mg | 5.1 | 6.2 | 0.0 mg | 11.4 | 4.8 | |  |
|  |  |  | PS |  | 4.4 | 5.0 |  | 11.4 | 4.8 | |  |
|  |  |  | PSC |  | 4.2 | 4.7 |  | 11.4 | 4.8 | |  |
| Folate | 100 μg, 130 μg,  150 μg | Female Optimum | None | 260.7 μg | 28.0 | 3.6 | 336.7 μg | 26.1 | 1.0 | |  |
|  |  |  | PS |  | 10.9 | 0.2 |  | 10.8 | 0.1 | |  |
|  |  |  | PSC |  | 5.0 | 0.0 |  | 5.0 | 0.0 | |  |
|  |  | Male Optimum | None | 129.2 μg | 69.9 | 44.6 | 133.6 μg | 77.5 | 42.9 | |  |
|  |  |  | PS |  | 46.6 | 15.7 |  | 59.6 | 15.7 | |  |
|  |  |  | PSC |  | 31.7 | 5.0 |  | 42.9 | 5.0 | |  |
| Vitamin B12 | 0.80 μg, 1.00 μg | Female Optimum | None | 0.0 μg | 0.5 | 0.0 | 0.0 μg | 0.0 | 0.0 | |  |
|  |  |  | PS |  | 0.5 | 0.0 |  | 0.0 | 0.0 | |  |
|  |  | Male Optimum | None | 0.0 μg | 0.5 | 0.0 | 0.0 μg | 0.0 | 0.0 | |  |
|  |  |  | PS |  | 0.5 | 0.0 |  | 0.0 | 0.0 | |  |
