# Supplementary material for: Projected effectiveness of mandatory industrial fortification of wheat flour, milk, and edible oil with multiple micronutrients among Mongolian adults
Source: PLoS One. 2018 Aug 2;13(8):e0201230. doi: 10.1371/journal.pone.0201230 (PMC6071971; doi:10.1371/journal.pone.0201230)
Supplement: S8 Table — "Optimal Level" represents the estimated concentration of nutrient needed to achieve a post-fortification intake deficiency prevalence of 5% (50% in the case of vitamin D) in a specific urban or rural area, season, and sex under maximum overage guidelines for processing, storage, and cooking (if the baseline prevalence is equal to or less than this percentage, the optimal level is set to 0). For comparison, published are reproduced from Table 1. The projected effect of each area-, season-, and sex-specific optimal level on the prevalence of intake deficiency (%<EAR) and over-sufficiency (%>UL) is modeled for both sexes in the same area and season under different overage guidelines. Shading indicates the extent of projected deficiency or over-sufficiency (0%: green; 50%: yellow; 100%: red). Abbreviations: IU (international unit; 40 IU = 1 μg), PS (overage for processing and storage losses), PSC (overage for processing, storage, and cooking losses). Iron and zinc losses in cooking flour products are negligible, therefore PSC overage for iron and zinc is not modeled. (DOCX) [file pone.0201230.s010.docx]

|  | | | | **Area and Season: Rural Summer** | | | | | **Area and Season: Rural Winter** | | | | | |
| --- | --- | --- | --- | --- | --- | --- | --- | --- | --- | --- | --- | --- | --- | --- |
| **Nutrient** | **Published Levels (per 100g of flour)** | **Modeled Guideline** | **Overage Guideline** | **Optimal Level (per 100g of flour)** | **%<EAR, Females** | **%>UL, Females** | **%<EAR, Males** | **%>UL, Males** | **Optimal Level (per 100g of flour)** | | **%<EAR, Females** | **%>UL, Females** | **%<EAR, Males** | **%>UL, Males** |
| Iron | 2.0 mg, 3.0 mg | Female | None | 2.6 mg | 5.4 | 0 | 0 | 2.3 | 1.3 mg | | 5.2 | 0 | 0 | 0 |
|  |  | Optimum | PS |  | 5.1 | 0 | 0 | 2.4 |  |  | 5 | 0 | 0 | 0 |
|  |  | Male | None | 0.0 mg | 18.7 | 0 | 0.5 | 0 | 0.0 mg | | 10.6 | 0 | 0 | 0 |
|  |  | Optimum | PS |  | 18.7 | 0 | 0.5 | 0 |  |  | 10.6 | 0 | 0 | 0 |
| Zinc | 3.0 mg, 4.0 mg | Female | None | 0.0 mg | 1.4 | 0 | 0.8 | 0.2 | 0.0 mg | | 1.9 | 0 | 0.5 | 2.5 |
|  |  | Optimum | PS |  | 1.4 | 0 | 0.8 | 0.2 |  |  | 1.9 | 0 | 0.5 | 2.5 |
|  |  | Male | None | 0.0 mg | 1.4 | 0 | 0.8 | 0.2 | 0.0 mg | | 1.9 | 0 | 0.5 | 2.5 |
|  |  | Optimum | PS |  | 1.4 | 0 | 0.8 | 0.2 |  |  | 1.9 | 0 | 0.5 | 2.5 |
| Vitamin A | 100 μg, 150 μg | Female | None | 151.8 μg | 13.3 | 1.5 | 22.4 | 4.6 | 267.0 μg | | 16.5 | 1.8 | 0 | 0 |
|  |  | Optimum | PS |  | 5.9 | 0.3 | 14.3 | 5.1 |  |  | 6.2 | 2 | 0 | 0 |
|  |  |  | PSC |  | 5 | 0.3 | 12.7 | 5.2 |  |  | 5 | 2.1 | 0 | 0 |
|  |  | Male | None | 210.5 μg | 5.8 | 0.3 | 13.8 | 5.1 | 105.5 μg | | 50.6 | 1.2 | 14 | 1.7 |
|  |  | Optimum | PS |  | 1.3 | 0.3 | 5.9 | 5.8 |  |  | 42.7 | 1.4 | 6.6 | 0.7 |
|  |  |  | PSC |  | 1 | 0.3 | 5 | 6 |  |  | 40.6 | 1.4 | 5 | 0.6 |
| Vitamin D | 55 IU | Female | None | 209.4 IU | 96.3 | 0 | 27 | 0 | 168.7 IU | | 77.6 | 0 | 28.4 | 0 |
|  |  | Optimum | PS |  | 50 | 0 | 11.6 | 0 |  |  | 50 | 0 | 7.6 | 0 |
|  |  |  | PSC |  | 50 | 0 | 11.6 | 0 |  |  | 50 | 0 | 7.6 | 0 |
|  |  | Male | None | 125.7 IU | 100 | 0 | 71.4 | 0 | 117.8 IU | | 98.3 | 0 | 86.7 | 0 |
|  |  | Optimum | PS |  | 100 | 0 | 50 | 0 |  |  | 89.1 | 0 | 50 | 0 |
|  |  |  | PSC |  | 100 | 0 | 50 | 0 |  |  | 89.1 | 0 | 50 | 0 |
| Niacin | 3.0 mg | Female | None | 0.9 mg | 6.2 | 0 | 2 | 11.1 | 1.1 mg | | 6.1 | 0.3 | 0.1 | 24.1 |
|  |  | Optimum | PS |  | 5.3 | 0 | 1.9 | 12.5 |  |  | 5.2 | 0.4 | 0.1 | 26.9 |
|  |  |  | PSC |  | 5 | 0 | 1.8 | 12.9 |  |  | 5 | 0.5 | 0 | 28 |
|  |  | Male | None | 0.0 mg | 11.6 | 0 | 3.4 | 6.1 | 0.0 mg | | 13 | 0.1 | 0.5 | 15.4 |
|  |  | Optimum | PS |  | 11.6 | 0 | 3.4 | 6.1 |  |  | 13 | 0.1 | 0.5 | 15.4 |
|  |  |  | PSC |  | 11.6 | 0 | 3.4 | 6.1 |  |  | 13 | 0.1 | 0.5 | 15.4 |
|  | | | | **Area and Season: Urban Summer** | | | | | **Area and Season: Urban Winter** | | | | | |
| **Nutrient** | **Published Levels (per 100g of flour)** | **Modeled Guideline** | **Overage Guideline** | **Optimal Level (per 100g of flour)** | **%<EAR, Females** | **%>UL, Females** | **%<EAR, Males** | **%>UL, Males** | **Optimal Level (per 100g of flour)** | **%<EAR, Females** | | **%>UL, Females** | **%<EAR, Males** | **%>UL, Males** |
| Iron | 2.0 mg, 3.0 mg | Female | None | 3.1 mg | 5.4 | 0.3 | 0 | 0.3 | 4.6 mg | 5.3 | | 1 | 0 | 6.9 |
|  |  | Optimum | PS |  | 5 | 0.4 | 0 | 0.4 |  | 5 | | 1.2 | 0 | 7.8 |
|  |  | Male | None | 0.0 mg | 16.8 | 0 | 0.2 | 0 | 0.0 mg | 22.2 | | 0 | 0 | 0.3 |
|  |  | Optimum | PS |  | 16.8 | 0 | 0.2 | 0 |  | 22.2 | | 0 | 0 | 0.3 |
| Zinc | 3.0 mg, 4.0 mg | Female | None | 0.0 mg | 2.1 | 0.1 | 0.5 | 0.2 | 0.0 mg | 0.3 | | 0 | 0.8 | 1 |
|  |  | Optimum | PS |  | 2.1 | 0.1 | 0.5 | 0.2 |  | 0.3 | | 0 | 0.8 | 1 |
|  |  | Male | None | 0.0 mg | 2.1 | 0.1 | 0.5 | 0.2 | 0.0 mg | 0.3 | | 0 | 0.8 | 1 |
|  |  | Optimum | PS |  | 2.1 | 0.1 | 0.5 | 0.2 |  | 0.3 | | 0 | 0.8 | 1 |
| Vitamin A | 100 μg, 150 μg | Female | None | 220.6 μg | 12.5 | 0.8 | 17.9 | 4.4 | 395.3 μg | 11.5 | | 0.5 | 0.3 | 7.6 |
|  |  | Optimum | PS |  | 5.6 | 1.2 | 7.9 | 5.2 |  | 5.6 | | 1 | 0 | 10 |
|  |  |  | PSC |  | 5 | 1.2 | 6.8 | 5.3 |  | 5 | | 1.1 | 0 | 10.5 |
|  |  | Male | None | 240.5 μg | 10.5 | 0.8 | 14.8 | 4.6 | 176.0 μg | 36.7 | | 0.2 | 11.7 | 9.7 |
|  |  | Optimum | PS |  | 4.4 | 1.3 | 5.8 | 5.5 |  | 26 | | 0.3 | 6.1 | 9.1 |
|  |  |  | PSC |  | 3.8 | 1.3 | 5 | 5.7 |  | 24.6 | | 0.3 | 5 | 9.1 |
| Vitamin D | 55 IU | Female | None | 178.8 IU | 73.4 | 0 | 38.8 | 0 | 207.1 IU | 72.5 | | 0 | 26.4 | 0 |
|  |  | Optimum | PS |  | 50 | 0 | 9.7 | 0 |  | 50 | | 0 | 8 | 0 |
|  |  |  | PSC |  | 50 | 0 | 9.7 | 0 |  | 50 | | 0 | 8 | 0 |
|  |  | Male | None | 129.4 IU | 92.7 | 0 | 85.3 | 0 | 135.0 IU | 95.1 | | 0 | 22.6 | 0 |
|  |  | Optimum | PS |  | 78.6 | 0 | 50 | 0 |  | 85.6 | | 0 | 50 | 0 |
|  |  |  | PSC |  | 78.6 | 0 | 50 | 0 |  | 85.6 | | 0 | 50 | 0 |
| Niacin | 3.0 mg | Female | None | 1.3 mg | 6.1 | 2.7 | 0.9 | 12.6 | 2.0 mg | 6.4 | | 0.9 | 0.5 | 22.6 |
|  |  | Optimum | PS |  | 5.3 | 3.2 | 0.6 | 13.9 |  | 5.3 | | 1.4 | 0.4 | 25.6 |
|  |  |  | PSC |  | 5 | 3.4 | 0.5 | 14.6 |  | 5 | | 1.6 | 0.3 | 26.9 |
|  |  | Male | None | 0.0 mg | 10.9 | 1.2 | 2.2 | 6.8 | 0.0 mg | 13.4 | | 0.2 | 2 | 12.7 |
|  |  | Optimum | PS |  | 10.9 | 1.2 | 2.2 | 6.8 |  | 13.4 | | 0.2 | 2 | 12.7 |
|  |  |  | PSC |  | 10.9 | 1.2 | 2.2 | 6.8 |  | 13.4 | | 0.2 | 2 | 12.7 |
