# Supplementary material for: Projected effectiveness of mandatory industrial fortification of wheat flour, milk, and edible oil with multiple micronutrients among Mongolian adults
Source: PLoS One. 2018 Aug 2;13(8):e0201230. doi: 10.1371/journal.pone.0201230 (PMC6071971; doi:10.1371/journal.pone.0201230)
Supplement: S1 File — (DOCX) [file pone.0201230.s011.docx]

**S1 File. Supplemental Methods**

Section A: Details on nutritional analysis of diet

Empirical recipes and dish yields [1] were generated for complex dishes by averaging information on their raw ingredient and cooked dish masses across all instances in which accurate measurement of these masses was feasible, applying ingredient yield factors [2,3] as needed when a dish yield could not be calculated. Nutrient composition of ingredients, single-ingredient food items, and complex dishes were compiled using a combination of unpublished locally-analyzed food composition data from the Mongolian University of Science and Technology and Mongolian Public Health Institute, food composition data from the United States and Germany [4,5], and entries from a combination of international reference tables previously compiled as part of a food composition table for Mongolian children [6]. Where applicable, dish yield and nutrient retention factors [2,7,8] were applied to calculate nutrient concentrations in cooked foods, as were adjustments for rendering compatible nutrient concentrations in borrowed and source food composition data with different moisture and fat contents [1].

Section B: Details on statistical programs

In this study, the Statistical Program to Assess Dietary Exposure (SPADE) [9] was used to correct repeated measurements of wheat flour, edible oil, and milk intake for within-person variance in order to estimate medians of the usual intake distributions of these fortification vehicles in different seasons and subgroups defined by urban/rural locality and sex. For this purpose, SPADE’s 1-part model was used, which is based on an earlier model by Waijers and colleagues [10] and is appropriate for dietary components consumed on a habitual basis (i.e. non-zero on almost every person-day of observation). In the 1-part model, only intake amounts are modeled rather than additionally modeling intake frequencies.

The 1-part model involves 3 steps as summarized by Dekkers and colleagues: Box-Cox transformation of observed daily intakes to obtain a normally-distributed intake distribution; modeling of transformed intakes as a function of age to estimate within- and between-person variance components with which to attenuate the variance of the observed intake distribution (an age-independent model is also allowed, but was not used in this study as there was no reason to do so; however, all ages were aggregated in presenting results of fortification vehicle intake); and back-transformation of the usual intake distribution to the response scale.

SPADE models may be run for males, females, or both sexes (in this study, males and females were modeled separately). SPADE allows inclusion of survey weights and population numbers to better represent the target population, and estimation of bootstrap confidence intervals to quantify uncertainty in the usual intake distributions (all of these features were used in this study).

The Intake Monitoring, Assessment, and Planning Program (IMAPP) [11] was used to project usual distributions of nutrient intake and the prevalence of intake deficiency and oversufficiency in different seasons and subgroups defined by urban/rural locality and sex, both before and after industrial fortification. Rather than model intake as a function of age as in SPADE, different IMAPP models are run for different age groups (in this study, age groups of adults were pooled given the homogeneity of their nutrient requirements). IMAPP implements the Iowa State University (ISU) method developed by Nusser and colleagues [12] and is an extension of the PC Software for Intake Distribution Estimation (PC-SIDE) engine.

The procedure involves the following steps as part of the PC-SIDE algorithm, as summarized by Nusser: adjustment of observed intake distributions for nuisance effects which may influence daily intake (in this study, the effect of day of the week was already accounted for by the survey weights used); adjustment of observed intakes so that they have a mean and variance of the first sample days’ (as measurement of this day’s intake is considered to be most accurate); application of survey weights; transformation of the observed intake distribution using power and grafted polynomial functions to achieve normality; estimation of the transformed distribution’s variance components and estimation in turn of the parameters of the usual intake distribution; and back-transformation of the usual intake distribution on the response scale.

In IMAPP, the PC-SIDE procedure is preceded by adjustment of nutrient intake on each person-day according to the daily intake of fortification vehicles and the concentration of fortificant to be added to each. Alternatively, the software can estimate the minimum fortificant concentration necessary to achieve a subgroup-specific prevalence of intake deficiency, by running a range of models bounded by minimum and maximum fortification levels until a desired prevalence is reached. After running each model, IMAPP applies the EAR cut-point method [13] to the usual intake distributions to estimate the prevalence of deficiency and over-sufficiency (in this study, age group- and sex-specific cutoffs were drawn from the Institute of Medicine Dietary Reference Intakes [14]); in the case of iron, the EAR cut-point method is inappropriate given iron’s lognormal requirement distribution in premenopausal women and therefore a full-probability approach is used instead [13].

Section C: Assumptions of fortification models

In modeling the effects of fortification, the following simplifying assumptions were made:

- A negligible fraction of wheat flour, edible oil, and milk is currently subject to voluntary industrial fortification in Mongolia (personal communication, June 2017: Enkhbileg G. (Executive Director of Dairy Unit, APU Company), Blüthner A (Director of Food Fortification and Partnerships, BASF), Leufgen A (General Manager, Stern Ingredients); this fraction was therefore not accounted for by the food composition data used in this study.
- Milk consumed in rural areas is entirely produced at home rather than industrially-processed, as supported by prevailing food consumption patterns in Mongolia [15]; rural areas are therefore impenetrable to industrial milk fortification.
- Actual concentrations of micronutrients present in fortification vehicles are equal to those stipulated by fortification and overage guidelines (described in subsequent sections). The validity of this assumption will depend on the closeness with which Mongolia’s upcoming fortification program is inspected and monitored.
- Industrial milk fortification only affects non-fermented liquid, dry, and condensed milk, and does not appreciably affect micronutrient concentrations in dairy products, including butter, cheese, cream, curds, and yogurt); conversely, industrial wheat flour fortification does affect products composed of wheat flour. While the validity of these assumptions will depend on technical considerations and the expansiveness of upcoming fortification legislation in Mongolia, their practicality is supported by inspection of national food composition data from the United States, a country in which fortification of wheat flour with B vitamins and iron is mandatory and fortification of milk with vitamin D is widespread, and in which wheat flour products are found to be rich in fortified nutrients while dairy products contain minimal vitamin D [5].
- Concentrations of fortificants found in wheat flour, edible oil, and milk imported to Mongolia are equal to those found in vehicles produced in and subject to fortification in Mongolia. The validity of this assumption will depend on the design and impact of future trade policy in Mongolia. Analysis of recent food balance data shows that wheat flour and flour products, and milk imports account for 11.0% and 1.7% of domestic supply, respectively, while 19.1% and 0% of imported flour and milk originates from countries known to fortify these vehicles (at least for domestic consumption) [16]. Furthermore, 100% of edible oil consumed in Mongolia is imported, some of which is fortified. In order for vehicles to meet Mongolian guidelines, it will be necessary for Mongolia to harmonize its international trade and domestic fortification guidelines by mandating that imported flour (particularly from Russia, its primary importer) and edible oil (which originates from many countries) are appropriately fortified.

Section D: Details on estimation of fortificant losses and overage factors

For edible oil and milk, processing and storage losses were estimated using nutrient- and vehicle-specific nutrient stability factors [17], cooking losses were estimated using nutrient- and vehicle-specific nutrient retention factors [2,8], and total losses were estimated by multiplying the former by the latter, being careful not to multiply factors which accounted for redundant steps in food preparation. Collected diet records did not consistently allow sufficient granularity to distinguish between boiled and un-boiled forms of milk, in part because the vast majority of consumed milk was observed to be boiled; for the purpose of assigning nutrient losses, all consumed liquid, unfermented milk was therefore assumed to have been boiled, an assumption not expected to materially affect the results of fortification models. Because the stability of nutrients in flour products is dependent on the type of product being considered (as is less the case with edible oil- and milk-containing products), and because different products are consumed in different proportions from one subgroup and season to another, nutrient-specific processing, storage, and cooking losses for flour were summarized for each of the 8 season-subgroups by assigning losses separately to each flour-product and taking an average weighted according to the observed mean fraction of total flour intake contributed by different flour products across all study participants in the season-subgroup.

Nutrient-specific overage factors were calculated for each food vehicle by taking the reciprocal of predicted nutrient losses attributable both to cooking alone and to processing, storage, and cooking, therefore providing overage factors for both processing and storage, and processing, storage, and cooking, respectively (S1 Table). In the case of flour, summer and winter means of subgroup-specific overage factors, weighted according to the size of each subgroup, were then estimated for each combination of nutrient, overage level, and season. An annualized average of summer and winter mean flour overage factors for each nutrient and overage level was calculated and tabulated along with the minimum and maximum of the 8 season-subgroup specific overage used to calculate each. For all nutrients, the range of estimated flour overage factors across season-subgroups is reasonably close to the average of weighted summer and winter means; the narrowest and widest ranges for processing, storage, and cooking are 1.418-1.448 (vitamin A) and 1.638-1.787 (thiamin), respectively.

**References**

1. Food and Agriculture Organization of the United Nations. FAO/INFOODS e-Learning Course on Food Composition Data. Rome: Food and Agriculture Organization of the United Nations; 2013. Available from: http://www.fao.org/elearning/#/elc/en/Course/FCD.
2. Bognár A. Tables on weight yield of food and retention factors of food constituents for the calculation of nutrient composition of cooked foods (dishes). Karlsruhe: Bundesforschungsanstalt für Ernährung; 2002.
3. Matthews RH, Garrison YJ. Food Yields Summarized by Different Stages of Preparation. United States Department of Agriculture Agricultural Research Service, Agriculture Handbook No. 102. Washington: United States Department of Agriculture; 1975.
4. Hartmann BM, Bell S, Vásquez-Caicedo AL. Bundeslebensmittelschlüssel II 3.1. Karlsruhe: Federal Research Centre for Nutrition and Food; 2005.
5. Haytowitz DB. USDA National Nutrient Database for Standard Reference, Release 24. Washington: United States Department of Agriculture; 2011.
6. Lander R, Enkhjargal TS, Batjargal J, Bolormaa N, Enkhmyagmar D, Tserendolgor U, et al. Poor dietary quality of complementary foods is associated with multiple micronutrient deficiencies during early childhood in Mongolia. Public Health Nutr. 2010 Sep;13(9):1304-13.
7. Nutrient Data Laboratory. USDA Table of Nutrient Retention Factors. Beltsville: United States Department of Agriculture Agricultural Research Service; 2007.
8. Showell BA, Williams JR, Duvall M, Howe JC, Patterson KY, Roseland JM, et al. USDA Table of Cooking Yields for Meat and Poultry. Baltimore: United States Department of Agriculture; 2012.
9. Dekkers AL, Verkaik-Kloosterman J, van Rossum CT, Ocké MC. SPADE, a new statistical program to estimate habitual dietary intake from multiple food sources and dietary supplements. J Nutr. 2014 Dec;144(12):2083-91.
10. Waijers PM, Dekkers AL, Boer JM, Boshuizen HC, van Rossum CT. The potential of AGE MODE, an age-dependent model, to estimate usual intakes and prevalences of inadequate intakes in a population. J Nutr 2006;136:2916–20.
11. World Health Organization. WHO Meeting on Estimating Appropriate Levels of Vitamins and Minerals for Food Fortification Programmes: The WHO Intake Monitoring, Assessment and Planning Program (IMAPP). Geneva: World Health Organization; 2010.
12. Nusser SM, Carriquiry AL, Dodd KW, Fuller WA. A Semiparametric Transformation Approach to Estimating Usual Daily Intake Distributions. J Am Stat Assoc. 1996 Dec;91(436):1440-9.
13. Institute of Medicine. DRI Dietary Reference Intakes: Applications in Dietary Assessment. Washington: National Academies Press; 2000.
14. Institute of Medicine. DRI Summary Tables. Washington: National Academies Press; 2014. Available from: http://www.nationalacademies.org/hmd/~/media/Files/Activity%20Files/Nutrition/DRI-Tables/5Summary%20TableTables%2014.pdf?la=en.
15. Food and Agriculture Organization of the United Nations. FAO/UNICEF/UNDP Report: Joint Food Security Assessment Mission to Mongolia. Ulaanbaatar: Food and Agriculture Organization of the United Nations; 2007.
16. Food and Agriculture Organization of the United Nations. FAOSTAT3 System. Rome: Food and Agriculture Organization of the United Nations; 2017. Available from: http://faostat3.fao.org.
17. DSM. Fortification Basics: Stability. Kaiseraugst: DSM Nutritional Products Ltd Nutrition Improvement Program; 2017. Report No.: 51610.
