# Supplementary material for: Projected effectiveness of mandatory industrial fortification of wheat flour, milk, and edible oil with multiple micronutrients among Mongolian adults
Source: PLoS One. 2018 Aug 2;13(8):e0201230. doi: 10.1371/journal.pone.0201230 (PMC6071971; doi:10.1371/journal.pone.0201230)
Supplement: S3 File — (DOCX) [file pone.0201230.s013.docx]

**S3 File. Policy Brief (English language)**

Despite steady and impressive progress in curbing wasting, stunting, and low birth weight, a persisting lack of dietary diversity underlies a high prevalence of multiple micronutrient deficiencies in Mongolia. In a nationwide, bi-annual survey of 320 healthy adults living in urban and rural areas of 7 national provinces and Ulaanbaatar, we found intake deficiencies of thiamin, folate, and vitamins A, D, and E to be highly prevalent, particularly among women. Micronutrient deficiencies in women of reproductive age pose a threat to their health as well as the health of their offspring during pregnancy and nursing, leading to potentially severe and permanent physical and cognitive deficits.

A large body of international evidence has demonstrated industrial fortification of staple foods with micronutrients, particularly when mandated by law and subject to appropriate inspection and monitoring, to be an effective, cost-saving, and safe strategy for improving nutrition in populations. At present, the only example of mandatory fortification in Mongolia is that of salt with iodine, which has proven to be an effective public health measure against goiter since its commencement in 1996.

Using data from our survey, we modeled the effects of different wheat flour, edible oil, and milk fortification guidelines on the prevalence of intake deficiency and over-sufficiency of 10 vitamins and minerals among 4 subpopulations of Mongolian adults in summer and winter, summarized in S2 Table. Results of our models indicate that flour fortification would be effective in reducing intake deficiencies of thiamin and folate, while the marginal benefit of fortification with iron and riboflavin would be smaller in part given these nutrients’ higher baseline consumption, and the benefit of fortification with zinc, niacin, and vitamin B12 is uncertain without data from children. Fortification of flour, oil, and milk with vitamins A, D, and E at levels suggested by international guidelines would substantially reduce vitamin A intake deficiency and would increase vitamin D intake considerably, with the greatest benefits elicited by wheat flour fortification and smaller benefits by additionally fortifying oil and milk.

These results strongly support the implementation of mandatory industrial fortification of wheat flour, edible oil, and milk with multiple micronutrients in Mongolia. We therefore provide the following policy guidance to assist the design and implementation of Mongolia’s national industrial fortification program, a bill for which is currently under legislative review by the Parliament:

- Fortification of wheat flour with thiamin, and folate is recommended at levels suggested by national guidelines published by the University of Science and Technology, riboflavin at half this level, and iron at the level published by the World Health Organization for countries consuming 300+ g/day of wheat flour. Overage should be applied to compensate for nutrient losses during food processing, storage, and cooking.
- Fortification of wheat flour and milk with vitamins A and D, and oil with vitamins A, D, and E is recommended. National guidelines for mandatory fortification of wheat flour and edible oil with vitamins A and E should be developed based on World Health Organization and DSM international guidelines, respectively, incorporating local cost and technical considerations. National guidelines for mandatory fortification of vitamins A and D in milk and vitamin D in wheat flour should also be developed by adapting initial guidance from existing regional standards published by the United States Department of Agriculture and Gulf Cooperation Council Standardization Organization, respectively.
- Fortification of wheat flour with zinc, niacin, and vitamin B12, while not supported by this analysis for adults, may prove beneficial for children. This should be affirmed by collection and analysis of food and nutrient intake data from children. Implementing national dietary surveillance should be a priority for monitoring and evaluation of fortification, and for public health in general in Mongolia
