# Supplementary material for: Projected effectiveness of mandatory industrial fortification of wheat flour, milk, and edible oil with multiple micronutrients among Mongolian adults
Source: PLoS One. 2018 Aug 2;13(8):e0201230. doi: 10.1371/journal.pone.0201230 (PMC6071971; doi:10.1371/journal.pone.0201230)
Supplement: S4 File — (DOCX) [file pone.0201230.s014.docx]

**S4 File. Policy brief and summary of baseline and projected prevalence of vitamin and mineral intake deficiencies (Mongolian language)**

**Аминдэм, эрдсийн хэрэглээний дутагдлын өнөөгийн байдал болон ирээдүйн чиг хандлага, бодлогын тойм**

Mонгол улсад хурц тураал, өсөлтийн хоцрогдол болон нярай бага жинтэй төрөх өвчлөлүүд тогтвортойгоор буурч байгаа боловч шим тэжээлийн олон-төрөлжилт хангалтгүй улмаас олон тооны бичил тэжээлийн дутагдлын тархалт өндөр хэвээр байна. Хот болон 7 аймагт амьдардаг нийт 320 насанд хүрэгчдийг хамруулсан үндэсний хэмжээний, жилд хоёр удаагийн давтамжтай судалгаанаас харахад ялангуяа эмэгтэйчүүдэд тиамин, фолийн хүчил, витамин А, Е болон Д-н дутагдал илэрчээ. Нөхөн үржихүйн насны эмэгтэйчүүдэд бичил тэжээлийн дутагдал үүсэх нь эхийн болон төрөх хүүхдийн эрүүл мэндэд эрсдэлтэй бөгөөд тухайлбал жирэмсний болон хөхүүл үеийн бичил тэжээлийн дутагдлаас хүүхдэд хүнд хэлбэрийн эргэн сэргэхгүй бие махбодын болон танин мэдэхүй гажгууд үүсдэг.

Хүнсний үйлдвэрлэлийн шатанд хүнс тэжээлийг бичил аминдэмээр баяжуулах бодлого нь ялангуяа хуулиар хамгаалагдаж хэрэгжилтэд сайтар хяналт тавьж чадвал хүн амын шим тэжээлийг дээшлүүлэх үр нөлөөтэй, санхүүгийн хувьд үр ашигтай, аюулгүй аргачлал болохыг олон тооны олон улсын судалгаанууд нотолсон байдаг. Одоогийн байдлаар Монгол улсад 1996 оноос хойш давсыг иодоор хуульчлан баяжуулж байгаа ба энэ нь бамбайн өвчинтэй тэмцэх нийгмийн эрүүл мэндийн том ололт амжилт болсон юм.

Бид судалгаандаа үндэслэн хэд хэдэн төрлийн буудайн гурил, хүнсний тос, сүүг баяжуулах удирдамжийн үр нөлөөг 10 төрлийн витамин, эрдсийн хомстол ба хэт илүүдлийн тархалтын төвшнөөр насанд хүрсэн хүмүүсийг баяжуулалтын өмнө ба дараа, өвөл болон зуны улиралд гэсэн 4 бүлэгт хувааж тооцоолон таамаглав (Xүснэгт). Уг загварын үр дүнд гурилыг бичил элементээр баяжуулснаар тиамин болон фолийн хүчлийн дутагдлыг багасгах боломжтой бөгөөд, харин төмөр болон рибофлавины суурь хэрэглээ өндөр байдаг тул баяжуулалтын үр нөлөө бага гэж үзсэн. Харин цайр, витамин В3, В12-н баяжуулалтын үр нөлөө нь хүүхдүүдээс авсан судалгаа байгаагүй учир тодорхойлох боломжгүй юм. Олон улсын зөвлөмжид тусгасан хэмжээгээр витамин А, Д, Е-р гурил, тос, сүү зэргийг баяжуулах нь витамин А-н дутагдлыг илэрхий багасгах, витамин Д-н хэрэглээг тодорхой хэмжээнд нэмэгдүүлэх боломжтой бөгөөд гурилыг баяжуулах нь хамгийн өндөр үр өгөөжтэй аргачлал бол сүү болон тосыг нэмж баяжуулах нь үр дүн бага ажээ.

Дээрх үр дүнгүүдээс харвал Монгол улсад олон төрлийн бичил элементийг үйлдвэрийн аргаар буудайн гурил, тос, сүүнд албадан баяжуулалтыг хэрэгжүүлэх шаардлагатай юм. Тиймээс бид Монголын үндэсний үйлдвэрийн баяжуулалт хөтөлбөрийн (одоогоор парламентад өргөн барьсан ба хууль боловсруулах зөвлөгөөнөөр хянагдаж байна) төлөвлөлт болон хэрэгжүүлэлтийг дэмжих дараах бодлогын удирдамжийг гарган хүргүүлж байна:

- Буудайн гурилыг тиамин, фолийн хүчлээр Монгол Улсын Шинжлэх Ухаан Технологийн Их Сургуулиас гаргасан удирдамжид заасны дагуу тодорхой төвшинд баяжуулах, рибофлавиныг уг хэмжээний хагастай тэнцэхүйц хэмжээгээр, төмрийг Дэлхийн Эрүүл Мэндийн Байгууллагын зөвлөмжийн дагуу өдөрт 300+ грам буудайн гурил хэрэглэдэг улс орнуудад зааварчилсан хэмжээгээр баяжуулна. Хүнсний боловсруулалт, хадгалалт, чаналтын үед алдагдаж болох тэжээлийн хэмжээг харгалзан үзэж баяжуулах бодисыг нэмж тооцоолно.
- Буудайн гурил болон сүүг витамин А ба Д-р, тосыг витамин А, Д, Е-р баяжуулах зөвлөмж хүргүүлж байна. Витамин А ба Е-р буудайн гурил болон тосыг албадан баяжуулах үндэсний зөвлөмжийг гаргахдаа Дэлхийн Эрүүл Мэндийн Байгууллага болон DSM-н олон улсын удирдамжийг тус тус үндэслэх ба улс орны техникийн болон эдийн засгийн чадамжийг харгалзан үзнэ. Мөн витамин А, Д-р сүүг; витамин Д-р буудайн гурилыг албадан баяжуулах үндэсний удирдамж боловсруулахдаа АНУ-ын Хөдөө аж ахуйн яамнаас нийтэлсэн бүс нутгийн эхний лавламж, Арабын булангийн хамтын ажиллагааны зөвлөлийн стандартчиллын байгууллагаас гарган мөрдөж буй стандартыг тус тус үндэслэнэ.
- Буудайн гурилыг цайр, ниацин, витамин В12-н албадан баяжуулалт хийн насанд хүрэгчдэд хэрэглэх нь дэмжигдээгүй боловч хүүхдэд үр нөлөөтэй байх боломжтой. Үүнийг хүүхдийн хоол тэжээлийн хэрэглээний мэдээлэл цуглуулан дүн шинжилгээ хийж баталгаажуулах хэрэгтэй. Хүнс баяжуулалтыг хянах болон үнэлэхэд, мөн нийгмийн эрүүл мэндийн салбарт нэн түрүүнд үндэсний хэмжээний хоол тэжээлийн тандалт судалгаа шаардлагатай байдаг.

**Хүснэгт: Монгол оронд витамин болон эрдсийн дутагдалд хүнсний баяжуулалт хийхийн өмнөх суурь үзүүлэлт болон дараа хүрэх таамагласан төвшин**

| **Бичил**  **Элемент** | **Баяжуулагч** | **Бүтээгдэхүүн** | **Төвшин**  **(/100**г) | **Баяжуулалтын**  **Өмнө (%<ТДХ)** | | **Баяжуулалтын**  **Дараа (%<ТДХ)** | |
| --- | --- | --- | --- | --- | --- | --- | --- |
|  |  |  |  | **Зун** | **Өвөл** | **Зун** | **Өвөл** |
| Төмөр | Төмрийн фумарат | Гурил | 2.0 мг | 9 | 10 | 4 | 5 |
| Цайр | Цайрын oксид |  | - | 1 | 1 | Б.Ш. | Б.Ш. |
| Тиамин | Тиамин мононитрат |  | 0.4 мг | 54 | 68 | 3 | 7 |
| Pибофлавин | Pибофлавин |  | 0.2 мг | 8 | 7 | 1 | 2 |
| Ниацин | Никотинамид |  | - | 7 | 8 | Б.Ш. | Б.Ш. |
| Фолат | Фолийн xүчил |  | 115 μг | 99 | 97 | 6 | 9 |
| Bитамин В12 | Цианокобаламин |  | - | 0 | 0 | Б.Ш. | Б.Ш. |
| Bитамин A | Pетинол пальмитат | Гурил | 117 μг | 53 | 59 | 9 | 17 |
|  |  | Тос | 900 μг |  |  |  |  |
|  |  | Сүү | 62 μг |  |  |  |  |
| Bитамин Д | Xолекальциферол | Гурил | 55 OУН | 100  (42 OУН /өдөр) | 100  (28 OУН  /өдөр) | 97  (213 OУН  /өдөр) | 99  (202 OУН  /өдөр) |
|  |  | Тос | 300 OУН |  |  |  |  |
|  |  | Сүү | 42 OУН |  |  |  |  |
| Bитамин E | Альфа токоферол | Тос | 10.7 мг | 99 | 99 | 95 | 96 |

Тэмдэглэл: Тодорхой хэмжээнд хүнс баяжуулалтыг хийхийн өмнөх суурь төвшин болон хүнс баяжуулалтын дараах үзүүлэлт гэдэг нь хүн амын дунд хоол хүнснээс авах шим тэжээлийн дутагдлын тархалтыг хувиар илэрхийлсэн (%<ТДХ: тооцоолсон дундаж хэмжээ) үзүүлэлт юм

Bитамин Д-ийн хувьд суурь ба таамагласан дундаж хэрэглээг (ОУН/өдөр) тусгасан. Уг үзүүлэлтүүд нь хүн амыг улсын хэмжээнд тооцсон ба хүнсний боловсруулалт, хадгалалт, болон чаналтаас хамааран хэмжээг нэмж тооцоолсон. Б.Ш. (Баяжуулах Шаардлагагүй): Монголд насанд хүрэгчдэд дээрх элементүүдийн дутагдлын тархалт харьцангуй бага эсвэл хэт илүүдэл үүсэх боломжтой тул тухайн хүнсийг баяжуулах шаардлагагүй гэж үзсэн. Гэвч хүүхдэд үр нөлөөг судалгаа хийх шаардлага тулгарч байна. OУН: oлон улсын нэгж (40 OУН = 1 μг).
