# Supplementary material for: Projected effectiveness of mandatory industrial fortification of wheat flour, milk, and edible oil with multiple micronutrients among Mongolian adults
Source: PLoS One. 2018 Aug 2;13(8):e0201230. doi: 10.1371/journal.pone.0201230 (PMC6071971; doi:10.1371/journal.pone.0201230)
Supplement: S5 File — (DOCX) [file pone.0201230.s015.docx]

**S5 File. External links and captions to interactive graphs of prevalence of vitamin A intake deficiency and over-sufficiency, and vitamin D intake deficiency and median intake under different overage guidelines (urban areas)**

**Prevalence of vitamin A intake deficiency under different overage guidelines (urban areas).** Available at: <http://rpubs.com/sbromage/Vitamin_A_intake_deficiency>. Figure shows the percentage of each urban subgroup's vitamin A intake lying below the subgroup-specific estimated average requirement (EAR) at baseline (Level 0) and projected under different fortification and overage guidelines. The x, y, and z axes span a range of lour, oil, and milk fortificant concentrations (in units of fortificant per 100 grams of vehicle), respectively, which correspond to the range of modeled levels. Shading indicates the extent of projected intake deficiency (lowest value: dark purple; highest value: bright yellow). See Methods and Table 1 for description of levels and references. When viewing a graph, place the cursor over each point to see more information, double-click legend items to cycle through population subgroups, click and drag to rotate the graph, and use the mouse wheel to zoom in and out. Note: graphs are only interpretable when one legend item is active at a time. Abbreviations: PS (overage for processing and storage losses), PSC (overage for processing, storage, and cooking losses).

**Prevalence of vitamin A over-sufficiency under different overage guidelines (urban areas).** Available at: <http://rpubs.com/sbromage/Vitamin_A_over-sufficiency>. Figure shows the percentage of each urban subgroup’s vitamin A intake lying above the subgroup-specific upper limit (UL) at baseline (Level 0) and projected under different fortification and overage guidelines. The x, y, and z axes span a range of flour, oil, and milk fortificant concentrations (in units of fortificant per 100 grams of vehicle), respectively, which correspond to the range of modeled levels. Shading indicates the extent of projected intake over-sufficiency (lowest value: dark purple; highest value: bright yellow). See Methods and Table 1 for description of levels and references. When viewing a graph, place the cursor over each point to see more information, double-click legend items to cycle through population subgroups, click and drag to rotate the graph, and use the mouse wheel to zoom in and out. Note: graphs are only interpretable when one legend item is active at a time. Abbreviations: PS (overage for processing and storage losses), PSC (overage for processing, storage, and cooking losses).

**Prevalence of vitamin D intake deficiency under different overage guidelines (urban areas).** Available at: <http://rpubs.com/sbromage/Vitamin_D_intake_deficiency>. Figure shows the percentage of each urban subgroup’s vitamin D intake lying below the subgroup-specific estimated average requirement (EAR) at baseline (Level 0) and projected under different fortification and overage guidelines. The x, y, and z axes span a range of flour, oil, and milk fortificant concentrations (in units of fortificant per 100 grams of vehicle), respectively, which correspond to the range of modeled levels. Shading indicates the extent of projected intake deficiency (lowest value: dark purple; highest value: bright yellow). See Methods and Table 1 for description of levels and references. When viewing a graph, place the cursor over each point to see more information, double-click legend items to cycle through population subgroups, click and drag to rotate the graph, and use the mouse wheel to zoom in and out. Note: graphs are only interpretable when one legend item is active at a time. Abbreviations: IU (international unit; 40 IU = 1 μg), PS (overage for processing and storage losses), PSC (overage for processing, storage, and cooking losses).

**Median intake of vitamin D under different overage guidelines (urban areas).** Available at: <http://rpubs.com/sbromage/Vitamin_D_median_intake>. Figure shows the median intake of vitamin D (IU/day) in each urban subgroup at baseline (Level 0) and projected under different fortification and overage guidelines. The x, y, and z axes span a range of flour, oil, and milk fortificant concentrations (in units of fortificant per 100 grams of vehicle), respectively, which correspond to the range of modeled levels. Shading indicates the magnitude of projected median intake (lowest value: dark purple; highest value: bright yellow). See Methods and Table 1 for description of levels and references. When viewing a graph, place the cursor over each point to see more information, double-click legend items to cycle through population subgroups, click and drag to rotate the graph, and use the mouse wheel to zoom in and out. Note: graphs are only interpretable when one legend item is active at a time. Abbreviations: IU (international unit; 40 IU = 1 μg), PS (overage for processing and storage losses), PSC (overage for processing, storage, and cooking losses).
