# Supplementary material for: Projected effectiveness of mandatory industrial fortification of wheat flour, milk, and edible oil with multiple micronutrients among Mongolian adults
Source: PLoS One. 2018 Aug 2;13(8):e0201230. doi: 10.1371/journal.pone.0201230 (PMC6071971; doi:10.1371/journal.pone.0201230)
Supplement: Supplementary file 14 [file pone.0201230.s001.docx]

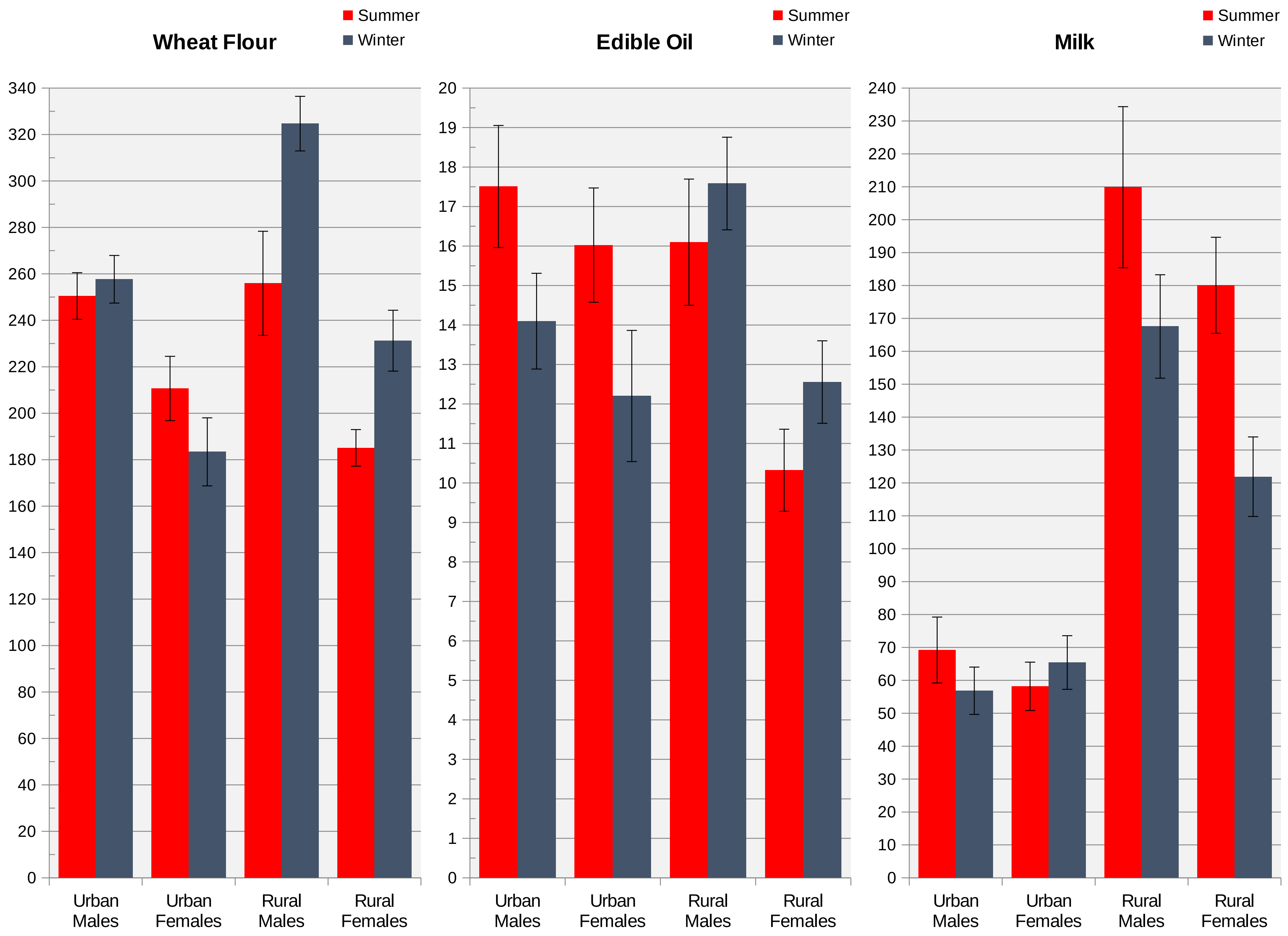
Wheat Flour
Summer
Winter
Edible Oil
Summer
Winter
Milk
Summer
Winter
Urban Males
Urban Females
Rural Males
Rural Females
