# Supplementary material for: Projected effectiveness of mandatory industrial fortification of wheat flour, milk, and edible oil with multiple micronutrients among Mongolian adults
Source: PLoS One. 2018 Aug 2;13(8):e0201230. doi: 10.1371/journal.pone.0201230 (PMC6071971; doi:10.1371/journal.pone.0201230)
Supplement: Supplementary file 15 [file pone.0201230.s002.docx]

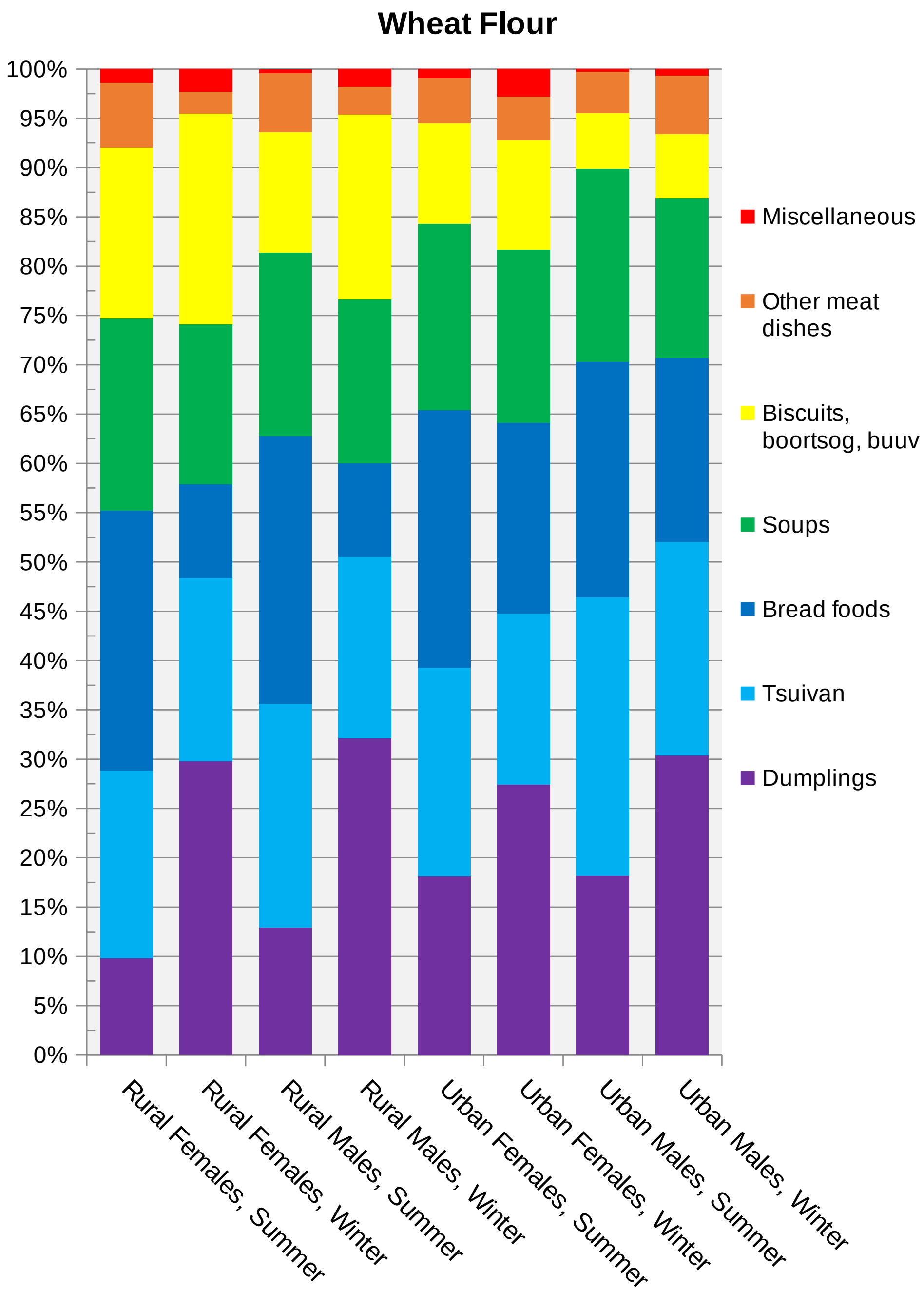
Wheat Flour
100%
95%
90%
85%
80%
75%
70%
65%
60%
55%
50%
45%
40%
35%
30%
25%
20%
15%
10%
5%
0%
Miscellaneous
Other meat dishes
Biscuits, boortsog, buuv
Soups
Bread foods
Tsuivan
Dumplings
Rural Females, Summer
Rural Females, Winter
Rural Males, Summer
Rural Males, Winter
Urban Females, Summer
Urban Females, Winter
Urban Males, Summer
Urban Males, Winter

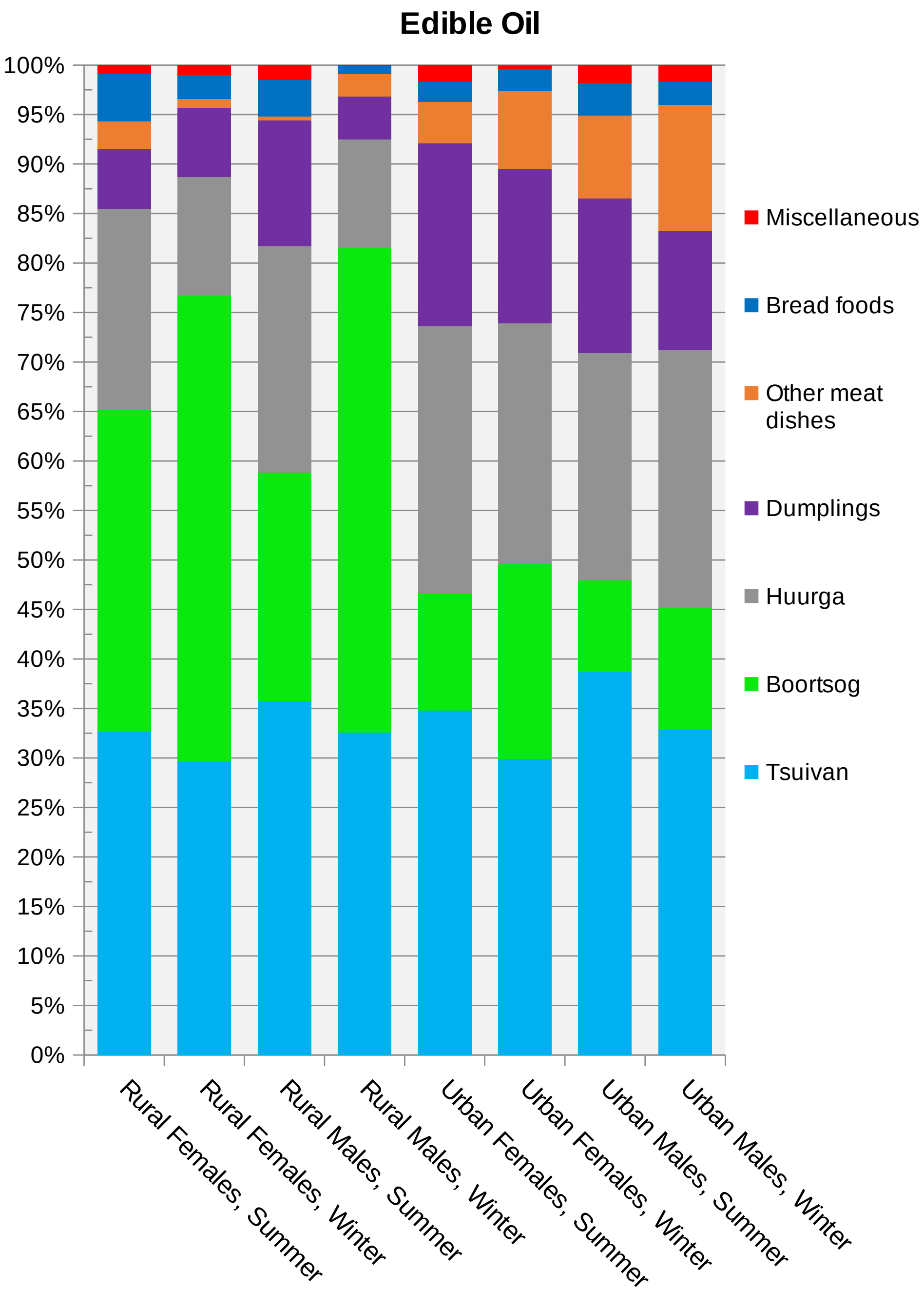
Edible Oil
100%
95%
90%
85%
80%
75%
70%
65%
60%
55%
50%
45%
40%
35%
30%
25%
20%
15%
10%
5%
0%
Miscellaneous
Bread foods
Other meat dishes
Dumplings
Huurga
Boortsog
Tsuivan
Rural Females, Summer
Rural Females, Winter
Rural Males, Summer
Rural Males, Winter
Urban Females, Summer
Urban Females, Winter
Urban Males, Summer
Urban Males, Winter
